# Complexity Analysis and Control of Output Competition in a Closed-Loop Supply Chain of Cross-Border E-Commerce Under Different Logistics Modes Considering Chain-to-Chain Information Asymmetry

**DOI:** 10.3390/e26121073

**Published:** 2024-12-09

**Authors:** Feng-Jie Xie, Lu-Ying Wen, Wen-Tian Cui, Xiao-Yang Shen

**Affiliations:** 1 School of Modern Posts, Xi’an University of Posts and Telecommunications, Xi’an 710061, China; 2 The School of Management, Xi’an Jiaotong University, Xi’an 710049, China

**Keywords:** supply chain, chain-to-chain competition, information sharing, logistics mode, discrete dynamics, entropy

## Abstract

To investigate the dynamic complexity of chain-to-chain output decisions in a closed-loop supply chain system of cross-border e-commerce (CBEC), this study decomposes the system into four product–market (PM) chains, based on the e-commerce platform’s information-sharing strategy and the manufacturer’s selected logistics mode (direct mail or bonded warehouse). By combining game theory with complex systems theory, discrete dynamic models for output competition among PM chains under four scenarios are constructed. The Nash equilibrium solution and stability conditions of the models are derived according to the principles of nonlinear dynamics. The stability of the model under the four scenarios, as well as the impacts of the initial output level and comprehensive tax rates on the stability and stability control of the system, are analyzed using numerical simulation methods. Our findings suggest that maintaining system stability requires controlling the initial output levels, the output adjustment speeds, and tariff rates to remain within specific thresholds. When these thresholds are exceeded, the entropy value of the model increases, and the system outputs decisions to enter a chaotic or uncontrollable state via period-doubling bifurcations. When the output adjustment speed of the four PM chains is high, the direct-mail logistics mode exhibits greater stability. Furthermore, under increased tariff rates for CBEC, the bonded warehouse mode has a stronger ability to maintain stability in system output decisions. Conversely, when the general import tax rate increases, the direct-mail mode demonstrates better stability. Regardless of the logistics mode, the information-sharing strategy can enhance the stability of system output decisions, while increased e-commerce platform commission rates tend to reduce stability. Interestingly, the use of a non-information-sharing strategy and the direct-mail logistics mode may be more conducive to increasing the profit levels of overseas manufacturers. Finally, the delayed feedback control method can effectively reduce the entropy value, suppress chaotic phenomena in the system, and restore stability to output decisions from a fluctuating state.

## 1. Introduction

In the era of globalization 2.0, competition within the global industrial and supply chain system is becoming increasingly intense, and cross-border e-commerce (CBEC) is gradually emerging as a key force in reshaping global industrial and supply chains [1,2]. According to data released by Statista, the value of the global CBEC market is expected to reach USD 7.9 trillion by 2030 [3]. In addition, relevant data from the “Annual report on the development of cross-border e-commerce in China (2024)” indicate that China’s CBEC import and export amounted to RMB 2.38 trillion in 2023, for a year-on-year increase of 15.6% [4]. This growth places higher demands on the stability of supply chains, particularly in mitigating systemic risks [5]. The dynamic evolution of competitive decisions within supply chain systems has a direct impact on market stability [6,7,8,9]. Compared to traditional supply chains, cross-border e-commerce (CBEC) supply chains face additional challenges, such as customs restrictions, logistical delays, and information asymmetry, all of which increase the complexity of competitive decision-making. Furthermore, environmental degradation and resource scarcity have prompted recycling enterprises to collaborate with e-commerce platforms, leading CBEC supply chain systems to exhibit closed-loop characteristics [10]. Therefore, studying the competitive decisions and dynamic evolution patterns associated with the closed-loop supply chain system of CBEC and providing critical scientific guidance for enterprises to make informed decisions are essential for maintaining the competitiveness of such systems in the global market.

In recent years, the expansion of global markets and the diversification of consumer demands have led to the emergence of various logistics modes [11,12,13]. Within the closed-loop supply chain system of CBEC, the primary logistics modes include the direct-mail mode and the bonded warehouse mode [14]. In the direct-mail mode, overseas suppliers ship products directly from overseas to consumers upon receiving orders. In contrast, the bonded warehouse mode allows overseas suppliers to pre-store products in domestic bonded warehouses, enabling rapid responses to consumer demand after orders are placed. Each mode has its advantages and disadvantages. The direct-mail mode incurs higher transaction costs but effectively reduces inventory risk, while the bonded warehouse mode minimizes transaction costs for overseas enterprises but poses risks of inventory shortages or surpluses. As a result, under different logistics modes, overseas suppliers must consider market demand, logistics costs, and inventory risks to formulate production decisions. These decisions influence the dynamic changes in output which, in turn, affect profits and market stability. Wang et al. [11], taking customer utility into account, explored the optimal strategy for retailers in the selection of cross-border logistics modes through a theoretical analysis and numerical simulations. Shao et al. [12] conducted a comparative analysis of consumer expectations under different logistics modes. Xiao et al. [13] quantitatively analyzed the industrial policies of CBEC and discussed the relevant laws and regulations for direct-mail and bonded warehouse logistics modes. Chen et al. [15] explored the impacts of different logistics modes on the product prices and transaction volumes of CBEC platform enterprises. The abovementioned studies on CBEC logistics modes have deeply discussed the differences between direct-mail and bonded warehouse logistics modes in CBEC systems but have neglected the phenomenon of information asymmetry that is common in CBEC systems.

Market information influences overseas suppliers’ assessment of inventory risks and transaction costs, thereby affecting their choice of logistics mode. CBEC platforms, with their extensive local market data and vast consumer information, enable overseas suppliers to better adjust their production decisions at each stage to capture a larger market share. However, on the other hand, sharing such data may lead CBEC platforms to lose part of their competitive advantage, potentially reducing the market share of their own products. Therefore, whether to share market information from the platform to enable overseas manufacturers to make accurate predictions is an important strategic decision for CBEC platforms [16,17]. Jiang et al. [18] found that, in a two-tier supply chain, retailers facing horizontal competition are motivated to exchange demand information when the intensity of competition is low, and this motivation is stronger when they procure from independent rather than monopolistic suppliers. Shang et al. [19] investigated the issue of information sharing in supply chains with two competing firms. Their research revealed that the motivation of retailers to share information strongly depends on non-linear production costs, the intensity of competition, and whether they can offer a contract to charge for the information. Lei et al. [20] studied ex-post-demand information sharing in a two-tier supply chain and discovered that retailers might voluntarily share low-demand information under certain conditions of product differentiation and demand scale. Hao et al. [21] showed that a sole supplier receives lower-quality information when adhering to a retail channel but experiences improved information quality when engaging in direct sales. Liu et al. [22] examined information-sharing strategies among multiple competing sellers distributing products on a retail platform and proposed a single-price mechanism to achieve an optimal information-sharing strategy. A comprehensive analysis of these studies indicates that they have predominantly focused on information-sharing strategies among domestic manufacturers or retailers within a single country or region. In contrast, research on information-sharing strategies of CBEC platforms in the cross-border context remains relatively scarce. Zhang et al. [23], by constructing a game theory model, explored the incentives for e-commerce platforms to share information with overseas manufacturers and further examined the role of information sharing under tax uncertainty. Zha et al. [24] developed a game-theoretic model to examine the information-sharing strategies of CBEC platforms and the logistics decisions of overseas suppliers. Their results show that, under conditions of intense competition and moderate market volatility, the platform is unlikely to share information and encourages suppliers to favor direct-mail logistics modes. Zhang et al. [25] further explored the impact of information sharing on logistics mode choices by constructing a multi-stage game model. Their study found that, with information sharing, the platform always favors the bonded warehouse mode. Without information sharing, the platform chooses between direct-mail and bonded warehouse modes based on tariff rates and market volatility. Both of these studies [23,24,25] analyze the issue from the perspective of competition among supply chain members within the CBEC system, focusing on how information sharing affects corporate decision-making and logistics mode selection.

With the advancement of information technology and the maturity of the market, competition among supply chain members within the same industry has transformed into chain-to-chain competition between supply chains [26,27,28]. McGuire et al. [26] were the first to propose a research framework for chain-to-chain competition in the context of traditional supply chains. Building on this foundation, Zhang et al. [27] introduced an economic model for chain-to-chain competition in traditional supply chains and presented a framework for its variational inequality. Feng et al. [28] developed a chain-to-chain output competition model for multi-national traditional supply chains, providing an in-depth analysis of the impact of trade policies on production equilibria. However, research focusing on the strategic choices and competitive decisions of CBEC supply chains from the perspective of chain-to-chain competition remains scarce. During the chain-to-chain competition process in CBEC supply chain systems, the output decisions of different supply chains are constantly adjusted and changed over time. Taking the supply chain of Apple smartphones as an example, the global shipment volume was approximately 80 million units in the fourth quarter of 2023, which was adjusted down to about 50 million units in the first quarter of 2024 [29]. Furthermore, driven by information technology and consumer environmental awareness, recyclers have gradually cooperated with CBEC platforms to jointly carry out trade-in services, and CBEC supply chain systems have also shifted towards a closed-loop model. For instance, in China, the CBEC platform JD.com has established a strategic partnership with the recycler GEM, and the used products recycled through JD.com are sold to GEM. Subsequently, GEM disassembles and reuses these obsolete products, selling the refined raw materials to overseas raw material suppliers such as the South Korean cathode material manufacturer ECOPRO [10]. ECOPRO further utilizes these raw materials to produce new products, which are ultimately supplied to internationally renowned manufacturers such as Samsung and Murata. Although the existing literature on competitive decision-making in CBEC supply chain systems [24,25] has analyzed the competitive decisions of the system under different logistics modes and information-sharing scenarios in depth, it has not taken into account the closed-loop characteristics of the CBEC supply chain system and has not studied competitive decision-making from the perspective of the supply chain. Therefore, the influence of chain-to-chain competition on the dynamic evolution of CBEC supply chain system decision-making has not been deeply explored, naturally, it is difficult to answer how to achieve the long-term stability of CBEC platforms and overseas manufacturers’ output decisions through scientific decision-making.

Motivated by the above discussions, this study focuses on the following research questions: (1) Considering the diversity of logistics modes, the closed-loop supply chain system of CBEC often exhibits a complex network structure. A critical challenge is determining how to extract the chain-to-chain competitive relationships between companies within this system. (2) How can a model describing the chain-to-chain multi-stage output competition of the closed-loop supply chain system of CBEC be established under different information-sharing strategies and logistics mode scenarios? (3) How can the Nash equilibrium points and stability conditions be determined? (4) What are the dynamic evolution patterns of production decisions in the closed-loop supply chain system of CBEC and how should companies make output decisions? When the dynamic evolution of production decisions exhibits instability, what strategies should be employed to control it? To answer the above questions, this study first constructs a closed-loop supply chain system of CBEC that includes overseas raw material suppliers and manufacturers, overseas CBEC platforms, domestic CBEC platforms, domestic logistics enterprises, and domestic recyclers and decomposes this system into four product–market (PM) chains based on the product flows. Second, based on the information sharing and logistics mode strategies of enterprises, discrete dynamic models of chain-to-chain competition for PM chains under four scenarios are established, where all PM chains play chain-to-chain Cournot games. Third, based on the principle of nonlinear dynamics, the Nash equilibrium points and the conditions required for stability under the four scenarios are obtained. Finally, through numerical simulation and analysis, the entropy and stability of system output decisions under the four scenarios, the impact of tariff rates on the system stability, the control method of system stability, and the sensitivity of parameters are explored.

The remainder of this paper is organized as follows. Section 2 describes the research problem. Section 3 establishes the discrete dynamic models. Section 4 provides a theoretical analysis of the models. Section 5 explores the system stability through numerical simulation. Finally, Section 6 presents the conclusions.

## 2. Problem Description

**Figure 1:**
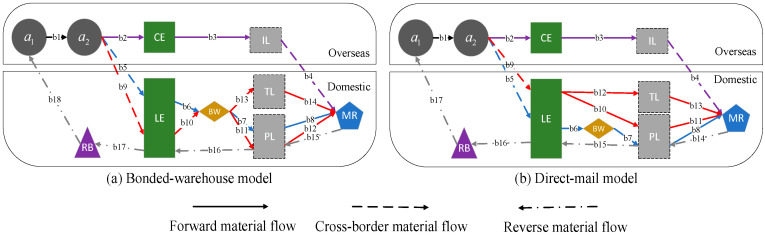
Closed-loop supply chain system of CBEC for product $P^{*}$ in the (**a**) bonded warehouse mode and (**b**) direct-mail mode.

This study develops a model of the closed-loop supply chain system of CBEC for a product $P^{*}$, including an overseas raw material supplier ($a_{1}$), overseas manufacturer ($a_{2}$), overseas CBEC platform (LE), domestic CBEC platform (CE), logistics companies (IL, TL and PL), a bonded warehouse (BW), and a recycler (RB), as shown in Figure 1. Let MR represent the domestic consumer market and $b_{m},m=1,2,\ldots ,18$ denote the material flow links between each company. The system includes two main logistics paths: the bonded warehouse mode and the direct-mail mode.

Bonded Warehouse Mode: The domestic CBEC platform LE pre-purchases products from overseas manufacturers $a_{2}$ and stores them in domestic bonded warehouses BW. After a consumer places an order, the product is delivered directly from the BW to the consumer via the platform’s own logistics PL or third-party logistics TL. For example, JD International sources Dyson vacuum cleaners from manufacturers in the U.K. and sells them through the “Dyson Overseas JD Self-operated Flagship Store”, with products delivered directly from the bonded warehouse to consumers via “JD Logistics”. Direct-Mail Mode: $a_{2}$ directly ships the product to the consumer where, after the product arrives, it is delivered to the consumer through PL or TL. For example, Dyson ships the product directly from the U.K. after a consumer places an order and, upon its arrival in China, the vacuum cleaner is delivered to Chinese consumers using JD Logistics or third-party logistics. Additionally, in a real CBEC supply chain system, $a_{2}$ can also deliver the product to the consumer through an overseas CBEC platform CE, utilizing international logistics IL.

To describe the chain-to-chain competition relationships within this system, this study refers to the concept of product–market (PM) chains, as proposed by Feng et al. [28], and decomposes the system into four PM chains. Each chain involves companies engaged in product procurement, production, transportation, recycling, and marketing; the target market; and the business activities connecting these companies. Let si,i=1,2,3,4 represent the four PM chains in the system. Under the bonded warehouse mode shown in Figure 1a, the four PM chains are s1 = $\left\{a_{1},a_{2},CE,IL,PL,LE,RB;b_{1},b_{2},b_{3},b_{4},b_{15},b_{16},b_{17},b_{18};MR\right\}$, s2 = $\left\{a_{1},a_{2},LE,BW,PL,RB;b_{1},b_{5},b_{6},b_{7},b_{8},b_{15},b_{16},b_{17},b_{18};MR\right\}$, s3 = $\left\{a_{1},a_{2},LE,BW,PL,RB;b_{1},b_{9},b_{10},b_{11},b_{12},b_{15},b_{16},b_{17},b_{18};MR\right\}$, and s4 = $\left\{a_{1},a_{2},LE,BW,TL,PL,RB;b_{1},b_{9},b_{10},b_{13},b_{14},b_{15},b_{16},b_{17},b_{18};MR\right\}$, where s1, s3, and s4 are the product $P^{*}$ sold by the overseas manufacturer $a_{2}$ on the e-commerce platform, while s2 represents self-operated products purchased by LE from $a_{2}$. Under the direct-mail mode shown in Figure 1b, the composition of PM chains s1 and s2 is the same as that of the bonded warehouse mode, while s3 = $\left\{a_{1},a_{2},LE,PL,RB;b_{1},b_{9},b_{10},b_{11},b_{14},b_{15},b_{16},b_{17};MR\right\}$ and s4 = $\left\{a_{1},a_{2},LE,TL,PL,RB;b_{1},b_{9},b_{12},b_{13},b_{14},b_{15},b_{16},b_{17};MR\right\}$. Similarly, s1, s3, and s4 denote the product $P^{*}$ sold by the overseas manufacturer $a_{2}$ on the e-commerce platform, while s2 represents self-operated products purchased by LE from $a_{2}$. It is important to note that different PM chains are subject to different taxes. If the product $P^{*}$ enters the market MR through the PM chain s1, consumers are required to pay the general trade import comprehensive tax, as stipulated by the local government. However, when $P^{*}$ enters the market through PM chains s2, s3, and s4, consumers should pay the corresponding CBEC import comprehensive tax. Generally, both the general trade import comprehensive tax $\chi_{c}$ and the CBEC import comprehensive tax $\chi_{e}$ consist of three components: import tariffs, value-added tax (VAT), and consumption tax [24].

The four PM chains vigorously compete for the market share of product $P^{*}$. Each PM chain is directed toward specific markets. If a node or a link is removed from the PM chain, the final product cannot be delivered. Therefore, the PM chains can be regarded as decision-making units in supply chain competition. To meet consumer demands in the market MR, the four PM chains compete in a Nash–Cournot pattern, where their strategy sets are the output levels of the four PM chains. In addition, supply chain competition in the real world is a dynamic process that exhibits adaptability and, so, we suppose that all PM chains exhibit bounded rationality.

## 3. Construction of Discrete Dynamic Model for the Closed-Loop Supply Chain System of CBEC

In the system shown in Figure 1, the domestic CBEC platform LE possesses extensive product and pricing information and faces a strategic decision: whether to share this information with the overseas manufacturer $a_{2}$. The non-information-sharing strategy of the domestic CBEC platform LE is denoted as Strategy N, while the information-sharing strategy is denoted as Strategy I. The bonded warehouse logistics mode of the overseas manufacturer $a_{2}$ is denoted as Mode B, while the direct-mail logistics mode is denoted as Mode D. Therefore, the information-sharing strategy of domestic CBEC platform LE and the logistics mode choices of the overseas manufacturer $a_{2}$ can lead to four competitive scenarios in the system shown in Figure 1: non-information sharing and bonded warehouse mode, denoted as Scenario NB; non-information-sharing strategy and direct-mail mode, denoted as Scenario ND; information sharing and bonded warehouse mode, denoted as Scenario IB; and information sharing and direct-mail mode, denoted as Scenario ID.

Referring to the economic model for supply chains in chain-to-chain competition proposed by Zhang and Feng et al. [27,28], this study develops a chain-to-chain competition economic model for the closed-loop supply chain system of CBEC. Furthermore, by innovatively integrating complex systems theory, multi-stage discrete dynamic models are derived to explore the dynamic evolution of the decision-making output within the system. The variables and symbols used in this paper are described in Table 1.

### 3.1. Non-Information-Sharing and Bonded Warehouse (NB)

#### 3.1.1. Inverse Demand Function of PM Chain

The four PM chains compete in a Nash–Cournot pattern to meet the demand of the consumer market. According to the supply chain economic model [27,28], the demand for the final product $P^{*}$ in the market is equal to the total output of the final product $P^{*}$ of all PM chains oriented to that market. Let the output of product $P^{*}$ on PM chain si be denoted as $x_{si,t}^{NB}$. Then, the total output oriented to market MR is $\sum_{i=1,2,3,4}x_{si,t}^{NB}$.

Considering that consumers have different preferences for products from different PM chains (channels) in a real CBEC supply chain system, let β(0<β<1) represent the channel substitutability between product $P^{*}$ sold by the overseas manufacturer $a_{2}$ through different PM chains (channels). In addition, the commissions charged by e-commerce platforms and the tariff costs incurred in cross-border trade are typically passed on to consumers, as reflected in the product price. Let $\varphi_{CE}$ and $\varphi_{LE}$ represent the commission rates of the e-commerce platform CE and LE, respectively, and let $\chi_{c}$ and $\chi_{e}$ represent the general import tax rate and the CBEC import tax rate, respectively. Based on these considerations, we model the inverse demand function of PM chain si in period t under the NB scenario as follows:

(1) $$\begin{array}{l} P_{s1,t}^{NB}=(E(u_{x})-x_{s1,t}^{NB}-\beta (x_{s2,t}^{NB}+x_{s3,t}^{NB}+x_{s4,t}^{NB}))(1+\varphi_{CE})(1+\chi_{c})\\ P_{s2,t}^{NB}=(u-x_{s2,t}^{NB}-\beta (x_{s1,t}^{NB}+x_{s3,t}^{NB}+x_{s4,t}^{NB}))(1+\chi_{e})\\ P_{s3,t}^{NB}=(E(u_{x})-x_{s3,t}^{NB}-\beta (x_{s1,t}^{NB}+x_{s2,t}^{NB}+x_{s4,t}^{NB}))(1+\varphi_{LE})(1+\chi_{e})\\ P_{s4,t}^{NB}=(E(u_{x})-x_{s4,t}^{NB}-\beta (x_{s1,t}^{NB}+x_{s2,t}^{NB}+x_{s3,t}^{NB}))(1+\varphi_{LE})(1+\chi_{e}) \end{array}$$

where u represents the market price of $P^{*}$ in market MR, and $E(u_{x})$ represents the expected market price of $P^{*}$ in market MR. The difference in market price between the PM chains is primarily due to the fact that the self-operated PM chain (s2) of the e-commerce platform LE can directly access market information and prices, while other PM chains (s1, s3, and s4) are at a greater market distance and are unable to obtain accurate information, thus having to rely on estimated market price expectations.

#### 3.1.2. The Cost for the Company Regarding Product $P^{*}$

To produce $x_{si,t}^{NB}$ units of product $P^{*}$, the overseas raw material supplier $a_{1}$ needs to supply the corresponding intermediate materials. For example, if $a_{1}$ manufactures cameras and participates in the PM chain for smartphones with a production volume of $x_{s1,t}^{NB}=100$, and one smartphone requires two cameras ($\rho_{a_{1}}=2$), the number of cameras that $a_{1}$ needs to provide for s1 is $\rho_{a_{1}}x_{s1,t}^{NB}=200$. Therefore, the material outputs of the four PM chains are as follows:

(2) $$\rho_{a_{1}}x_{si,t}^{NB},i=1,2,3,4$$

In the bonded warehouse mode, due to the fact that PM chains s2, s3, and s4 store product $P^{*}$ in bonded warehouses with a quantity of $x_{si,t}^{NB}$ before receiving orders, the actual sales volume of product $P^{*}$ in each period t is often less than $x_{si,t}^{NB}$. Let $\Delta_{si},i=2,3,4$ represent the actual sales ratio of product $P^{*}$ for PM chain si in each period t. Then, the actual sales volume for product $P^{*}$ from PM chains s2, s3, and s4 in period t would be $\Delta_{si}x_{si,t}^{NB},i=2,3,4$. The remaining inventory for PM chains s2, s3, and s4 in period t is denoted as $(1-\Delta_{si})x_{si,t}^{NB},i=2,3,4$, and this inventory will not need to be produced in the next period t+1. Then, the material output of $a_{1}$ for s2, s3, and s4 can be revised as follows:

(3) $$\rho_{a_{1}}(x_{si,t}^{NB}-(1-\Delta_{si})x_{si,t-1}^{NB}),i=2,3,4$$

Moreover, in the closed-loop supply chain shown in Figure 1, the recycler RB will collect the used product $P^{*}$ from the previous period t−1 via the domestic CBEC platform LE and sell it to the overseas raw material supplier $a_{1}$. After processing, $a_{1}$ will not only produce new products using new materials but also re-manufacture the recycled products. Finally, the new products and re-manufactured products will be sold downstream to the overseas manufacturer $a_{2}$. Let υ represent the recycling rate for product $P^{*}$ in the market and η represent the recyclable ratio of the recycled products. Given the bonded warehouse, the quantities of product $P^{*}$ available for recycling from the four PM chains are $x_{s1,t}^{NB}$, and $\Delta_{si}x_{si,t}^{NB},i=2,3,4$, then the recyclable output $f_{RBsi,t}^{NB}$ for the re-manufactured product by RB of the four PM chains is as follows:

(4) $$f_{RBsi,t}^{NB}=\left\{ \begin{array}{l} \eta \upsilon x_{si,t-1}^{NB},i=1\\ \eta \upsilon \Delta_{si}x_{si,t-1}^{NB},i=2,3,4 \end{array}\right.$$

In period t, the actual output $\rho_{a_{1}}x_{si,t}^{NB},i=1,2,3,4$ of the overseas raw material supplier $a_{1}$ in PM chain s1 includes the production output of new materials $f_{a_{1}s1,t}^{NB+}=\rho_{a_{1}}(x_{s1,t}^{NB}-\eta \upsilon x_{s1,t-1}^{NB})$ and the production of re-manufactured materials $f_{a_{1}s1,t}^{NB-}=\rho_{a_{1}}\eta \upsilon x_{s1,t-1}^{NB}$. Similarly, in the actual output $f_{a_{1}si,t}^{NB}=\rho_{a_{1}}(x_{si,t}^{NB}-(1-\Delta_{si})x_{si,t-1}^{NB}),i=2,3,4$ for PM chains s2, s3, and s4, the production of new materials $f_{a_{1}si,t}^{NB+}=\rho_{a_{1}}(x_{si,t}^{NB}-(1-\Delta_{si})x_{si,t-1}^{NB}-\eta \upsilon \Delta_{si}x_{si,t-1}^{NB}),i=2,3,4$ and re-manufactured materials $f_{a_{1}si,t}^{NB-}=\rho_{a_{1}}\eta \upsilon \Delta_{si}x_{si,t-1}^{NB},i=2,3,4$ are allocated to PM chains s2, s3, and s4. Therefore, the total material output of $a_{1}$ for the four PM chains can be expressed as follows:

(5) $$f_{a_{1}si,t}^{NB}=f_{a_{1}si,t}^{NB+}+f_{a_{1}si,t}^{NB-}=\left\{ \begin{array}{l} \rho_{a_{1}}(x_{s1,t}^{NB}-\eta \upsilon x_{s1,t-1}^{NB})+\rho_{a_{1}}\eta \upsilon x_{s1,t-1}^{NB},i=1\\ \rho_{a_{1}}(x_{si,t}^{NB}-(1-\Delta_{si})x_{si,t-1}^{NB}-\eta \upsilon \Delta_{si}x_{si,t-1}^{NB})+\rho_{a_{1}}\eta \upsilon \Delta_{si}x_{si,t-1}^{NB},i=2,3,4 \end{array}\right.$$

where $f_{a_{1}si,t}^{NB}$ represents the output of $a_{1}$ in PM chain si, $f_{a_{1}si,t}^{NB+}$ denotes the output of new materials by $a_{1}$ in PM chain si, and $f_{a_{1}si,t}^{NB-}$ indicates the output of re-manufactured products by $a_{1}$ in PM chain si.

After completing production, the overseas raw material supplier $a_{1}$ supplies materials $f_{a_{1}si,t}^{NB}$ to the overseas manufacturer $a_{2}$. Let $\rho_{a_{2}}$ represent the amount of intermediate materials that $a_{2}$ provides for one unit of $P^{*}$. Then, the material output of $a_{2}$ in the four PM chains is as follows:

(6) $$f_{a_{1}si,t}^{NB}=\left\{ \begin{array}{l} \rho_{a_{2}}x_{si,t}^{NB},i=1\\ \rho_{a_{2}}(x_{si,t}^{NB}-(1-\Delta_{si})x_{si,t-1}^{NB}),i=2,3,4 \end{array}\right.$$

Subsequently, $a_{2}$ introduces the completed final product $P^{*}$ into the market MR through its participation in the e-commerce platform CE. As a result, $x_{si,t}^{NB}$ units of the final product $P^{*}$ are introduced via CE, while $x_{s2,t}^{NB}-(1-\Delta_{s2})x_{s2,t-1}^{NB}$ and $x_{si,t}^{NB}-(1-\Delta_{si})x_{si,t-1}^{NB},i=3,4$ units of $P^{*}$ are supplied to or introduced onto the e-commerce platform LE for market entry. Due to the residual inventory in bonded warehouses, LE not only operates $x_{si,t}^{NB}-(1-\Delta_{si})x_{si,t-1}^{NB}$ units of $P^{*}$ from PM chains s2, s3, and s4, but also manages the remaining inventory from period t−1, represented as $(1-\Delta_{si})x_{si,t-1}^{NB}$ units of $P^{*}$. Thus, in period t, LE needs to operate a total of $x_{si,t}^{NB}$ units of $P^{*}$. In addition, as LE is involved in the recycling of $P^{*}$ for the four PM chains in market MR, it also needs to manage a recycled quantity of $\upsilon x_{si,t-1}^{NB},i=1,2,3,4$ units of $P^{*}$. Therefore, in period t, the quantities of $P^{*}$ that the CBEC platforms CE and LE need to operate in each PM chain are as follows:

(7) $$f_{CEs1,t}^{NB}=x_{s1,t}^{NB};f_{LEsi,t}^{NB}=\left\{ \begin{array}{l} \upsilon x_{si,t-1}^{NB},i=1\\ x_{si,t}^{NB}+\upsilon \Delta_{si}x_{si,t-1}^{NB},i=2,3,4 \end{array}\right.$$

Although the bonded warehouse BW must continue to store the remaining quantity of $P^{*}$ from period t−1, denoted as $(1-\Delta_{si})x_{si,t-1}^{NB},i=2,3,4$, only $x_{si,t}^{NB}-(1-\Delta_{si})x_{si,t-1}^{NB},i=2,3,4$ units of new product $P^{*}$ are replenished into BW during each period t. Consequently, the quantity of $P^{*}$ that BW needs to store in each period t is effectively equivalent to the production output $x_{si,t}^{NB},i=2,3,4$ of $P^{*}$ on the PM chain. Then, the quantity of $P^{*}$ that BW requires for storage in each PM chain is expressed as follows:

(8) $$f_{BWsi,t}^{NB}=x_{si,t}^{NB},i=2,3,4$$

Upon receiving consumer orders, the $P^{*}$ stored in the bonded warehouse BW is delivered to the consumers by logistics companies. In the supply chain system illustrated in Figure 1a, as only $\Delta_{si}x_{si,t}^{NB},i=2,3,4$ units of $P^{*}$ can be sold at each stage, the domestic third-party logistics company TL only needs to handle $\Delta_{si}x_{si,t}^{NB},i=2,3,4$ units of $P^{*}$ from PM chain s4 during each period t. The platform logistics company PL is responsible for handling $\Delta_{si}x_{si,t}^{NB},i=2,3$ units of $P^{*}$ from PM chains s2 and s3. In addition, due to cooperation between the e-commerce platform and the recycler, PL must also manage the transportation of recovered $P^{*}$ from all four PM chains to the recycler RB, with $\upsilon \Delta_{si}x_{si,t-1}^{NB},i=2,3,4$ units from s1 and $\upsilon \Delta_{si}x_{si,t-1}^{NB},i=2,3,4$ units from s2, s3, and s4. Thus, during period t, the quantities of $P^{*}$ that IL, TL, and PL are required to handle in each PM chain are as follows:

(9) $$f_{ILs1,t}^{NB}=x_{s1,t}^{NB};f_{TLs4,t}^{NB}=\Delta_{s4}x_{s4,t}^{NB};f_{PLsi,t}^{NB}=\left\{ \begin{array}{l} \upsilon x_{si,t-1}^{NB},i=1\\ \Delta_{si}x_{si,t}^{NB}+\upsilon \Delta_{si}x_{si,t-1}^{NB},i=2,3\\ \upsilon \Delta_{si}x_{si,t-1}^{NB},i=4 \end{array}\right.$$

Let $r_{a_{1}}$ represent the marginal production cost for the overseas raw material supplier $a_{1}$ to produce new materials for PM chain $a_{1}$, $r_{a_{1}}^{-}$ represent the marginal production cost for producing re-manufactured materials, and $r_{a_{2}}$ represent the marginal production cost for the overseas manufacturer $a_{2}$ to produce product $P^{*}$ for PM chain si. Considering that, as the production scale of $a_{1}$ and $a_{2}$ expands, unit average costs may increase due to economies of scale, it is appropriate to use a quadratic nonlinear cost function as the production cost function [30]. Therefore, the production cost functions for $a_{1}$ and $a_{2}$ are given by the following:

(10) $$\begin{array}{l} C_{a_{1}si,t}^{NB}=r_{a_{1}}{f_{a_{1}si,t}^{NB+}}^{2}+r_{a_{1}}^{-}{f_{a_{1}si,t}^{NB-}}^{2},i=1,2,3,4\\ C_{a_{2}si,t}^{NB}=r_{a_{2}}{f_{a_{2}si,t}^{NB}}^{2},i=1,2,3,4 \end{array}$$

Let $r_{RB}$ and $h_{RB}$ represent the marginal re-manufacturing and processing costs for the recycler RB regarding waste $P^{*}$ for PM chain si. Considering that the re-manufacturing of products from the recycler is similar to the production process for product $P^{*}$, unit average costs may also increase with scale, so a quadratic nonlinear function is used for re-manufacturing costs. For waste treatment, as the process is relatively simple, a linear function is assumed for the cost. The recycling operational cost $C_{RBsi,t}^{NB}$ for RB can be expressed as follows:

(11) $$C_{RBsi,t}^{NB}=\left\{ \begin{array}{l} r_{RB}{f_{RBsi,t}^{NB}}^{2}+h_{RB}(1-\eta )\upsilon x_{si,t-1}^{NB},i=1\\ r_{RB}{f_{RBsi,t}^{NB}}^{2}+h_{RB}(1-\eta )\upsilon \Delta_{si}x_{si,t-1}^{NB},i=2,3,4 \end{array}\right.$$

As the operational processes of $P^{*}$ for the e-commerce platform and the logistics companies are relatively simple, we assume that the costs associated with product operations, bonded warehouse storage, and logistics operations are linear functions. Then, the operational costs for the e-commerce platforms CE and LE and the logistics companies IL, TL, and PL regarding product $P^{*}$ in PM chain si, as well as the storage cost for the bonded warehouse BW, are expressed as follows:

(12) $$\begin{array}{l} C_{CEs1,t}^{NB}=r_{CE}f_{CEs1,t}^{NB};C_{LEsi,t}^{NB}=r_{LE}f_{LEsi,t}^{NB},i=1,2,3,4\\ C_{ILs1,t}^{NB}=r_{IL}f_{ILs1,t}^{NB};C_{TLs4,t}^{NB}=r_{TL}f_{TLs4,t}^{NB};C_{PLsi,t}^{NB}=f_{PLsi,t}^{NB},i=1,2,3,4\\ C_{BWsi,t}^{NB}=r_{BW}f_{BWsi,t}^{NB},i=2,3,4 \end{array}$$

#### 3.1.3. The Transaction Cost for the Links Regarding $P^{*}$

In period t, the output levels of the front-end node (company) for each link $b_{m}$ in PM chain si determine the material flow of the link. For instance, company $a_{1}$, as the front-end node of link $b_{1}$, produces a quantity $\rho_{a_{1}}(x_{s2,t}^{NB}-(1-\Delta_{s2})x_{s2,t-1}^{NB})$ of material for PM chain s2. This quantity will be sold to company $a_{2}$ and generate a material flow of $\rho_{a_{1}}(x_{s2,t}^{NB}-(1-\Delta_{s2})x_{s2,t-1}^{NB})$ in link $b_{1}$ for PM chain s2. Therefore, the material flow in the link $b_{m},m=1,2,3,4,15,16,17,18$ for PM chain s1 is given by the following:

(13) $$\begin{array}{l} l_{b_{1}s1,t}^{NB}=f_{a_{1}s1,t}^{NB};l_{b_{2}s1,t}^{NB}=f_{a_{2}s1,t}^{NB};l_{b_{3}s1,t}^{NB}=f_{CEs1,t}^{NB};l_{b_{4}s1,t}^{NB}=f_{ILs1,t}^{NB};\\ l_{b_{15}s1,t}^{NB}=l_{b_{16}s1,t}^{NB}=l_{b_{17}s1,t}^{NB}=\upsilon x_{s1,t-1}^{NB};l_{b_{18}s1,t}^{NB}=f_{RBs1,t}^{NB} \end{array}$$

It is important to note that, for PM chains s2, s3, and s4, although the domestic CBEC platform operates a quantity $x_{si,t}^{NB}+\upsilon \Delta_{si}x_{si,t-1}^{NB},i=2,3,4$ of $P^{*}$ each period t, links $b_{6}$ and $b_{10}$ only contain forward material flows $x_{si,t}^{NB},i=2,3,4$, while link $b_{17}$ only contains the reverse material flow $\upsilon \Delta_{si}x_{si,t-1}^{NB},i=2,3,4$. Furthermore, under the NB scenario, the actual sales quantity of $P^{*}$ (denoted as $\Delta_{si}x_{si,t}^{NB}$) is not equal to the operational quantity of $P^{*}$ at the bonded warehouse BW (denoted as $x_{si,t}^{NB}$). Therefore, the material flow in links $b_{7},b_{11},b_{13}$ for PM chains s2, s3, and s4 is $\Delta_{si}x_{si,t}^{NB}$, which is not equal to the quantity $f_{BWsi,t}^{NB}=x_{si,t}^{NB},i=2,3,4$ operated by the front-end node company BW for product $P^{*}$. The material flow in links $b_{m},m=1,5,6,7,8,15,16,17,18$ for PM chain s2 can be obtained with the specific expressions detailed in Appendix B.

Similarly, the material flow in links $b_{m},m=1,9,10,11,12,15,16,17,18$ and $b_{m},m=1,9,10,13,14,15,16,17,18$ for PM chains s3 and s4 can be derived with the specific expressions provided in Appendix B.

In the links where material flows occur, certain transaction costs are incurred, including distribution and transportation costs. Considering that expanding the delivery scale of the link may lead to increasing average transaction costs due to the larger material flows, a non-economic scale effect may occur. Therefore, a quadratic nonlinear cost function is used for the transaction cost function. Let $r_{b_{m}}$ represent the marginal transaction cost of link $b_{m}$. Then, the transaction cost $C_{b_{m}si,t}^{NB}$ of link $b_{m}$ in each PM chain is given by the following:

(14) $$C_{b_{m}si,t}^{NB}=r_{b_{m}}{l_{b_{m}si,t}^{NB}}^{2}$$

#### 3.1.4. Discrete Dynamic Model of the System 

According to the supply chain economics model [27,28], the total cost of the PM chain si includes the production, operation, storage, and re-manufacturing costs of all companies in this PM chain for product $P^{*}$, as well as the total transaction costs of the links. Under the NB scenario, the total cost $C_{si,t}^{NB}$ for the four PM chains can be expressed as follows:

(15) $$\begin{array}{ll} C_{s1,t}^{NB} & =C_{a_{1}s1,t}^{NB}+C_{a_{2}s1,t}^{NB}+C_{CEs1,t}^{NB}+C_{ILs1,t}^{NB}+C_{PLs1,t}^{NB}+C_{LEs1,t}^{NB}+C_{RBs1,t}^{NB}+C_{b_{1}s1,t}^{NB}+C_{b_{2}s1,t}^{NB}+C_{b_{3}s1,t}^{NB}+C_{b_{4}s1,t}^{NB}+\\ & C_{b_{15}s1,t}^{NB}+C_{b_{16}s1,t}^{NB}+C_{b_{17}s1,t}^{NB}+C_{b_{18}s1,t}^{NB}\\ & =\gamma_{1}{x_{s1,t}^{NB}}^{2}-\gamma_{2}x_{s1,t-1}^{NB}x_{s1,t}^{NB}+\gamma_{3}{x_{s1,t-1}^{NB}}^{2}+\gamma_{4}x_{s1,t}^{NB}+\gamma_{5}x_{s1,t-1}^{NB}\\ C_{s2,t}^{NB} & =C_{a_{1}s2,t}^{NB}+C_{a_{2}s2,t}^{NB}+C_{LEs2,t}^{NB}+C_{PLs2,t}^{NB}+C_{BWs2,t}^{NB}+C_{RBs2,t}^{NB}+C_{b_{1}s2,t}^{NB}+C_{b_{5}s2,t}^{NB}+C_{b_{6}s2,t}^{NB}+C_{b_{7}s2,t}^{NB}+C_{b_{8}s2,t}^{NB}+\\ & C_{b_{15}s2,t}^{NB}+C_{b_{16}s2,t}^{NB}+C_{b_{17}s2,t}^{NB}+C_{b_{18}s2,t}^{NB}\\ & =\gamma_{6}{x_{s2,t}^{NB}}^{2}-\gamma_{7}x_{s2,t-1}^{NB}x_{s2,t}^{NB}+\gamma_{8}{x_{s2,t-1}^{NB}}^{2}+\gamma_{9}x_{s2,t}^{NB}+\gamma_{10}x_{s2,t-1}^{NB}\\ C_{s3,t}^{NB} & =C_{a_{1}s3,t}^{NB}+C_{a_{2}s3,t}^{NB}+C_{LEs3,t}^{NB}+C_{PLs3,t}^{NB}+C_{BWs3,t}^{NB}+C_{RBs3,t}^{NB}+C_{b_{1}s3,t}^{NB}+C_{b_{9}s3,t}^{NB}+C_{b_{10}s3,t}^{NB}+C_{b_{11}s3,t}^{NB}+C_{b_{12}s3,t}^{NB}+\\ & C_{b_{15}s3,t}^{NB}+C_{b_{16}s3,t}^{NB}+C_{b_{17}s3,t}^{NB}+C_{b_{18}s3,t}^{NB}\\ & =\gamma_{11}{x_{s3,t}^{NB}}^{2}-\gamma_{12}x_{s3,t-1}^{NB}x_{s3,t}^{NB}+\gamma_{13}{x_{s3,t-1}^{NB}}^{2}+\gamma_{14}x_{s3,t}^{NB}+\gamma_{15}x_{s3,t-1}^{NB}\\ C_{s4,t}^{NB} & =C_{a_{1}s4,t}^{NB}+C_{a_{2}s4,t}^{NB}+C_{LEs4,t}^{NB}+C_{TLs4,t}^{NB}+C_{BWs4,t}^{NB}+C_{PLs4,t}^{NB}+C_{RBs4,t}^{NB}+C_{b_{1}s4,t}^{NB}+C_{b_{9}s4,t}^{NB}+C_{b_{10}s4,t}^{NB}+C_{b_{13}s4,t}^{NB}+\\ & C_{b_{14}s4,t}^{NB}+C_{b_{15}s4,t}^{NB}+C_{b_{16}s4,t}^{NB}+C_{b_{17}s4,t}^{NB}+C_{b_{18}s4,t}^{NB}\\ & =\gamma_{16}{x_{s4,t}^{NB}}^{2}-\gamma_{17}x_{s4,t-1}^{NB}x_{s4,t}^{NB}+\gamma_{18}{x_{s4,t-1}^{NB}}^{2}+\gamma_{19}x_{s4,t}^{NB}+\gamma_{20}x_{s4,t-1}^{NB} \end{array}$$

where the concrete expressions of $\gamma_{1}-\gamma_{20}$ are detailed in Appendix A.

The profit of PM chain si is the difference between its revenue and cost. Thus, the profit of the four PM chains can be expressed as follows:

(16) $$\left\{ \begin{array}{l} \pi_{s1,t}^{NB}=P_{s1,t}^{NB}x_{s1,t}^{NB}-C_{s1,t}^{NB}\\ \pi_{si,t}^{NB}=P_{si,t}^{NB}\Delta_{si}x_{si,t}^{NB}-C_{si,t}^{NB},i=2,3,4 \end{array}\right.$$

where the concrete expression of Formula (16) is detailed in Appendix C.

Supply chain competition in the real world is a dynamic process. We suppose that PM chain si exhibits bounded rationality and its output decisions are adjusted for the next period based on the current decisions and the corresponding marginal profit; that is, when the marginal profit of PM chain si is estimated to be positive (negative) in period t, si will increase (decrease) the decision value in the following period [6,7]. Let $\omega_{si}^{NB}$ denote the output adjustment speed of si under the NB scenario. Then, the adjustment process of the output decisions of si under the NB scenario can be expressed as follows:

(17) $$x_{si,t+1}^{NB}=x_{si,t}^{NB}+\omega_{si}^{NB}x_{si,t}^{NB}\frac{\partial \pi_{si,t}^{NB}}{\partial x_{si,t}^{NB}},i=1,2,3,4$$

Setting $x_{si,t-1}^{NB}=q_{si,t}^{NB},i=1,2,3,4$ in Equation (17), the discrete dynamic system model for the chain-to-chain game of the closed-loop supply chain system of CBEC under the NB scenario can be expressed as follows:

(18) $$\begin{array}{l} x_{s1,t+1}^{NB}=x_{s1,t}^{NB}+\omega_{s1}^{NB}x_{s1,t}^{NB}((E(u_{x})-2x_{s1,t}^{NB}-\beta (x_{s2,t}^{NB}+x_{s3,t}^{NB}+x_{s4,t}^{NB}))(1+\varphi_{CE})(1+\chi_{c})-2\gamma_{1}x_{s1,t}^{NB}+\gamma_{2}q_{s1,t}^{NB}-\gamma_{4})\\ x_{s2,t+1}^{NB}=x_{s2,t}^{NB}+\omega_{s2}^{NB}x_{s2,t}^{NB}((u-2x_{s2,t}^{NB}-\beta (x_{s1,t}^{NB}+x_{s3,t}^{NB}+x_{s4,t}^{NB}))(1+\chi_{e})\Delta_{s2}-2\gamma_{6}x_{s2,t}^{NB}+\gamma_{7}q_{s2,t}^{NB}-\gamma_{9})\\ x_{s3,t+1}^{NB}=x_{s3,t}^{NB}+\omega_{s3}^{NB}x_{s3,t}^{NB}((E(u_{x})-2x_{s3,t}^{NB}-\beta (x_{s1,t}^{NB}+x_{s2,t}^{NB}+x_{s4,t}^{NB}))(1+\varphi_{LE})(1+\chi_{e})\Delta_{s3}-2\gamma_{11}x_{s3,t}^{NB}+\gamma_{12}q_{s3,t}^{NB}-\gamma_{14})\\ x_{s4,t+1}^{NB}=x_{s4,t}^{NB}+\omega_{s4}^{NB}x_{s4,t}^{NB}((E(u_{x})-2x_{s4,t}^{NB}-\beta (x_{s1,t}^{NB}+x_{s2,t}^{NB}+x_{s3,t}^{NB}))(1+\varphi_{LE})(1+\chi_{e})\Delta_{s4}-2\gamma_{16}x_{s4,t}^{NB}+\gamma_{17}q_{s4,t}^{NB}-\gamma_{19})\\ q_{s1,t+1}^{NB}=x_{s1,t}^{NB}\\ q_{s2,t+1}^{NB}=x_{s2,t}^{NB}\\ q_{s3,t+1}^{NB}=x_{s3,t}^{NB}\\ q_{s4,t+1}^{NB}=x_{s4,t}^{NB} \end{array}$$

### 3.2. Non-Information-Sharing and Direct-Mail (ND)

#### 3.2.1. Inverse Demand Function of PM Chain

As a change in the logistics mode does not affect the functional relationship between the market price and the production quantity in each PM chain, the composition of the inverse demand functions for each PM chain under the ND scenario is the same as that under the NB scenario, that is, $P_{si,t}^{NB}=P_{si,t}^{ND},i=1,2,3,4$.

#### 3.2.2. The Cost for the Company Regarding Product $P^{*}$

Under the ND scenario, the companies participating in PM chains s1 and s2 are the same as those under the NB scenario. Therefore, the cost composition of each company in s1 and s2 is the same as that for the companies under the NB mode, that is, $C_{\varepsilon si,t}^{ND}=C_{\varepsilon si,t}^{NB},i=1,2$.

The introduction of the direct-mail mode has a significant impact on the companies participating in PM chains s3 and s4. Under the direct-mail mode, the actual transaction volume in period t for s3 and s4 is equal to the production quantity $x_{si,t}^{ND},i=3,4$ which, in turn, leads to changes in the production quantity of the companies in PM chains s3 and s4 for each period t. Specifically, under the ND scenario, the recyclable output $f_{RBsi,t}^{ND},i=3,4$ for the recycler RB of PM chains s3 and s4 is as follows:

(19) $$f_{RBsi,t}^{ND}=\eta \upsilon x_{si,t-1}^{ND},i=3,4$$

Due to the absence of a bonded warehouse, the overseas raw material supplier $a_{1}$ and the overseas manufacturer $a_{2}$ in PM chains s3 and s4 will not reduce production based on the inventory surplus from the previous period. Therefore, in period t, the actual material output ($\rho_{a_{1}}x_{si,t}^{ND},i=3,4$) of the overseas raw material supplier $a_{1}$ in the PM chain can be expressed as follows: the quantity of new materials produced for PM chains s3 and s4 is $f_{a_{1}si,t}^{ND+}=\rho_{a_{1}}(x_{si,t}^{ND}-\eta \upsilon x_{si,t-1}^{ND}),i=3,4$, and the quantity of re-manufactured materials is $f_{a_{1}si,t}^{ND-}=\rho_{a_{1}}\eta \upsilon x_{si,t-1}^{ND},i=3,4$. Thus, the material output of $a_{1}$ in PM chains s3 and s4 can be further expressed as:

(20) $$f_{a_{1}si,t}^{ND}=f_{a_{1}si,t}^{ND+}+f_{a_{1}si,t}^{ND-}=\rho_{a_{1}}(x_{si,t}^{ND}-\eta \upsilon x_{si,t-1}^{ND})+\rho_{a_{1}}\eta \upsilon x_{si,t-1}^{ND},i=3,4$$

After $a_{1}$ completes production, it sells $f_{a_{1}si,t}^{ND}$ units of material to the overseas manufacturer $a_{2}$. Therefore, the material output for $a_{2}$ in PM chains s3 and s4, for the production of $x_{si,t}^{NB}$ units of $P^{*}$, is as follows:

(21) $$f_{a_{1}si,t}^{ND}=\rho_{a_{2}}x_{si,t}^{ND},i=3,4$$

Therefore, the e-commerce platform LE needs to operate $x_{si,t}^{NB}$ units of $P^{*}$ in PM chains s3 and s4, as well as a recycled quantity of $\upsilon x_{si,t-1}^{NB},i=1,2,3,4$ units of $P^{*}$. Thus, in period t, the CBEC platform CE needs to operate the following quantity of $P^{*}$ in PM chains s3 and s4:

(22) $$f_{LEsi,t}^{ND}=x_{si,t}^{ND}+\upsilon x_{si,t-1}^{ND},i=3,4$$

Subsequently, the local third-party logistics company TL and the platform logistics company PL deliver $x_{si,t}^{ND}$ units to consumers. In addition, PL also needs to handle a recycled quantity of $\upsilon x_{si,t-1}^{NB}$ units of $P^{*}$. Therefore, in period t, the quantities of $P^{*}$ that TL and PL need to handle in PM chains s3 and s4 are as follows:

(23) $$f_{ILsi,t}^{ND}=x_{si,t}^{ND},i=3,4;f_{PLsi,t}^{ND}=x_{si,t}^{ND}+\upsilon x_{si,t-1}^{NB},i=3,4$$

Thus, the production cost functions $C_{a_{1}si,t}^{ND},i=3,4$ and $C_{a_{2}si,t}^{Nd},i=3,4$ for $a_{1}$ and $a_{2}$ in PM chains s3 and s4 are, respectively, as follows:

(24) $$\begin{array}{l} C_{a_{1}si,t}^{ND}=r_{a_{1}}{f_{a_{1}si,t}^{ND+}}^{2}+r_{a_{1}}^{-}{f_{a_{1}si,t}^{ND-}}^{2},i=3,4\\ C_{a_{2}si,t}^{ND}=r_{a_{2}}{f_{a_{2}si,t}^{ND}}^{2},i=3,4 \end{array}$$

The recycling operation cost $C_{RBsi,t}^{NB},i=3,4$ for the recycler RB in PM chains s3 and s4 can be expressed as follows:

(25) $$C_{RBsi,t}^{ND}=r_{RB}{f_{RBsi,t}^{ND}}^{2}+h_{RB}(1-\eta )\upsilon x_{si,t-1}^{ND},i=3,4$$

#### 3.2.3. The Transaction Cost for the Links Regarding $P^{*}$

Under the ND scenario, as the product quantities produced and operated by the companies participating in PM chains s1 and s2 are consistent with those under the NB scenario, the composition of the link costs for $b_{m},m=1,2,3,4,5,6,7,8,14,15,16,17$ in PM chains s1 and s2 is also the same as that under the NB mode; that is, $C_{b_{m}s1,t}^{ND}=C_{b_{m}s1,t}^{NB},m=1,2,3,4,15,16,17,18$, $C_{b_{m}s2,t}^{ND}=C_{b_{m}s2,t}^{NB},m=1,2,5,6,7,8,15,16,17,18$.

For links $b_{m}$ in PM chains s3 and s4, under the direct-mail mode, the material flow is equal to the production quantity of $P^{*}$ by the terminal node (company). Therefore, the material flow for links $b_{m},m=1,9,10,11,14,15,16,17$ in PM chain s3 can be expressed as follows:

(26) $$l_{b_{1}s3,t}^{ND}=f_{a_{1}s3,t}^{ND};l_{b_{9}s3,t}^{ND}=l_{b_{10}s3,t}^{ND}=l_{b_{11}s3,t}^{ND}=x_{s3,t}^{ND};l_{b_{14}s3,t}^{ND}=l_{b_{15}s3,t}^{ND}=l_{b_{16}s3,t}^{ND}=\upsilon x_{s3,t-1}^{NB};l_{b_{17}s3,t}^{ND}=f_{RBs3,t}^{ND}$$

The material flow of links $b_{m}$ in PM chain s4 is as follows:

(27) $$l_{b_{1}s4,t}^{ND}=f_{a_{1}s4,t}^{ND};l_{b_{9}s4,t}^{ND}=l_{b_{12}s4,t}^{ND}=l_{b_{13}s4,t}^{ND}=x_{s4,t}^{ND};l_{b_{14}s4,t}^{ND}=l_{b_{15}s4,t}^{ND}=l_{b_{16}s4,t}^{ND}=\upsilon x_{s4,t-1}^{NB};l_{b_{17}s4,t}^{ND}=f_{RBs4,t}^{ND}$$

Thus, the transaction costs $C_{b_{m}si,t}^{NB}$ for link $b_{m}$ in PM chains s3 and s4 are as follows:

(28) $$C_{b_{m}si,t}^{NB}=r_{b_{m}}{l_{b_{m}si,t}^{NB}}^{2},i=3,4$$

#### 3.2.4. Discrete Dynamic Model of the System

The total cost of PM chain si includes the production and operational costs of all companies in this PM chain for the product $P^{*}$, as well as the total transaction costs of the links. Under the ND scenario, the total costs for PM chains s1 and s2 are the same as those under the NB scenario; that is, $C_{s1,t}^{ND}=C_{s1,t}^{NB}$ and $C_{s2,t}^{ND}=C_{s2,t}^{NB}$. The total cost for PM chains s3 and s4 is as follows:

(29) $$\begin{array}{ll} C_{s3,t}^{ND} & =C_{a_{1}s3,t}^{ND}+C_{a_{2}s3,t}^{ND}+C_{LEs3,t}^{ND}+C_{PLs3,t}^{ND}+C_{RBs3,t}^{ND}+C_{b_{1}s3,t}^{ND}+C_{b_{9}s3,t}^{ND}+C_{b_{10}s3,t}^{ND}+\\ & C_{b_{11}s3,t}^{ND}+C_{b_{14}s3,t}^{ND}+C_{b_{15}s3,t}^{ND}+C_{b_{16}s3,t}^{ND}+C_{b_{17}s3,t}^{ND}\\ & =\gamma_{21}{x_{s3,t}^{ND}}^{2}-\gamma_{22}x_{s3,t-1}^{ND}x_{s3,t}^{ND}+\gamma_{23}{x_{s3,t-1}^{ND}}^{2}+\gamma_{24}x_{s3,t}^{ND}+\gamma_{25}x_{s3,t-1}^{ND}\\ C_{s4,t}^{ND} & =C_{a_{1}s4,t}^{ND}+C_{a_{2}s4,t}^{ND}+C_{LEs4,t}^{ND}+C_{TLs4,t}^{ND}+C_{PLs4,t}^{ND}+C_{RBs4,t}^{ND}+C_{b_{1}s4,t}^{ND}+C_{b_{9}s4,t}^{ND}+\\ & C_{b_{12}s4,t}^{ND}+C_{b_{13}s4,t}^{ND}+C_{b_{14}s4,t}^{ND}+C_{b_{15}s4,t}^{ND}+C_{b_{16}s4,t}^{ND}+C_{b_{17}s4,t}^{ND}\\ & =\gamma_{26}{x_{s4,t}^{ND}}^{2}-\gamma_{22}x_{s4,t-1}^{ND}x_{s4,t}^{ND}+\gamma_{23}{x_{s4,t-1}^{ND}}^{2}+\gamma_{27}x_{s4,t}^{ND}+\gamma_{25}x_{s4,t-1}^{ND} \end{array}$$

where the concrete expressions of $\gamma_{21}-\gamma_{27}$ are detailed in Appendix A.

The profit for PM chain si is the difference between its revenue and cost. Thus, the profits of PM chains s1 and s2 are consistent with those under the NB scenario; that is, $\pi_{s1,t}^{ND}=\pi_{s1,t}^{NB}$, $\pi_{s3,t}^{ND}=\pi_{s3,t}^{NB}$. The profits for PM chains s3 and s4 are as follows:

(30) $$\pi_{si,t}^{ND}=P_{si,t}^{ND}x_{si,t}^{ND}-C_{s3,t}^{ND},i=3,4$$

where the concrete expression of Formula (30) is detailed in Appendix C.

Based on the adjustment process $x_{si,t+1}^{ND}=x_{si,t}^{ND}+\omega_{si}^{ND}x_{si,t}^{ND}\frac{\partial \pi_{si,t}^{ND}}{\partial x_{si,t}^{ND}},i=1,2,3,4$ of the output decisions of the PM chain si, and by setting $x_{si,t-1}^{ND}=q_{si,t}^{ND},i=1,2,3,4$, the discrete dynamic system model of the chain-to-chain game of the closed-loop supply chain system of CBEC under the ND scenario can be expressed as:

(31) $$\begin{array}{l} x_{s1,t+1}^{ND}=x_{s1,t}^{ND}+\omega_{s1}^{ND}x_{s1,t}^{ND}((E(u_{x})-2x_{s1,t}^{ND}-\beta (x_{s2,t}^{ND}+x_{s3,t}^{ND}+x_{s4,t}^{ND}))(1+\varphi_{CE})(1+\chi_{c})-2\gamma_{1}x_{s1,t}^{ND}+\gamma_{2}q_{s1,t}^{ND}-\gamma_{4})\\ x_{s2,t+1}^{ND}=x_{s2,t}^{ND}+\omega_{s2}^{ND}x_{s2,t}^{ND}((u-2x_{s2,t}^{ND}-\beta (x_{s1,t}^{ND}+x_{s3,t}^{ND}+x_{s4,t}^{ND}))(1+\chi_{e})\Delta_{s2}-2\gamma_{6}x_{s2,t}^{ND}+\gamma_{7}q_{s2,t}^{ND}-\gamma_{9})\\ x_{s3,t+1}^{ND}=x_{s3,t}^{ND}+\omega_{s3}^{ND}x_{s3,t}^{ND}((E(u_{x})-2x_{s3,t}^{ND}-\beta (x_{s1,t}^{ND}+x_{s2,t}^{ND}+x_{s4,t}^{ND}))(1+\varphi_{LE})(1+\chi_{e})-2\gamma_{21}x_{s3,t}^{ND}+\gamma_{22}q_{s3,t}^{ND}-\gamma_{24})\\ x_{s4,t+1}^{ND}=x_{s4,t}^{ND}+\omega_{s4}^{ND}x_{s4,t}^{ND}((E(u_{x})-2x_{s4,t}^{ND}-\beta (x_{s1,t}^{ND}+x_{s2,t}^{ND}+x_{s3,t}^{ND}))(1+\varphi_{LE})(1+\chi_{e})-2\gamma_{26}x_{s4,t}^{ND}+\gamma_{22}q_{s4,t}^{ND}-\gamma_{27})\\ q_{s1,t+1}^{ND}=x_{s1,t}^{ND}\\ q_{s2,t+1}^{ND}=x_{s2,t}^{ND}\\ q_{s3,t+1}^{ND}=x_{s3,t}^{ND}\\ q_{s4,t+1}^{ND}=x_{s4,t}^{ND} \end{array}$$

### 3.3. Information Sharing and Bonded Warehouse (IB)

#### 3.3.1. Inverse Demand Function of PM Chain

Under the information-sharing strategy, the overseas manufacturer $a_{2}$ can obtain the market price u of product $P^{*}$ in market MR. Therefore, the expected market price $E(u_{x})$ in the inverse demand functions of PM chains s1, s3, and s4 can be replaced with u. At this point, the inverse demand functions of the four PM chains are as follows:

(32) $$\begin{array}{l} P_{s1,t}^{IB}=(u-x_{s1,t}^{IB}-\beta (x_{s2,t}^{IB}+x_{s3,t}^{IB}+x_{s4,t}^{IB}))(1+\varphi_{CE})(1+\chi_{c})\\ P_{s2,t}^{IB}=(u-x_{s2,t}^{IB}-\beta (x_{s1,t}^{IB}+x_{s3,t}^{IB}+x_{s4,t}^{IB}))(1+\chi_{e})\\ P_{s3,t}^{IB}=(u-x_{s3,t}^{IB}-\beta (x_{s1,t}^{IB}+x_{s2,t}^{IB}+x_{s4,t}^{IB}))(1+\varphi_{LE})(1+\chi_{e})\\ P_{s4,t}^{IB}=(u-x_{s4,t}^{IB}-\beta (x_{s1,t}^{IB}+x_{s2,t}^{IB}+x_{s3,t}^{IB}))(1+\varphi_{LE})(1+\chi_{e}) \end{array}$$

#### 3.3.2. The Cost for the Company Regarding Product $P^{*}$

As the information-sharing strategy only affects the estimate of the price of product $P^{*}$ for the overseas manufacturer $a_{2}$ and does not impact the production or operational costs of each company regarding $P^{*}$, the composition of the production and operational costs for each company under the IB scenario are the same as those under the NB scenario; that is, $C_{\varepsilon si,t}^{IB}=C_{\varepsilon si,t}^{NB},i=1,2,3,4$.

#### 3.3.3. The Transaction Cost for the Links Regarding $P^{*}$

The material flows and transaction costs for each link $b_{m}$ of the PM chain are determined by the production quantity of $P^{*}$ from the companies participating in the PM chain. Therefore, under the IB scenario, the composition of the transaction costs in $b_{m}$ is the same as that under the NB scenario; that is, $C_{b_{m}si,t}^{IB}=C_{b_{m}si,t}^{NB},i=1,2,3,4$.

#### 3.3.4. Discrete Dynamic Model of the System

The composition of the total costs for each PM chain is the same as that under the NB scenario $C_{si,t}^{IB}=C_{si,t}^{NB},i=1,2,3,4$.

The profit of PM chain si is the difference between its revenue and cost; thus, under the IB scenario, the profits of four PM chains are as follows:

(33) $$\left\{ \begin{array}{l} \pi_{s1,t}^{IB}=P_{s1,t}^{IB}x_{s1,t}^{IB}-C_{s1,t}^{IB}\\ \pi_{si,t}^{IB}=P_{si,t}^{IB}\Delta_{si}x_{si,t}^{IB}-C_{si,t}^{IB},i=2,3,4 \end{array}\right.$$

where the concrete expression of Formula (33) is detailed in Appendix C.

Based on the adjustment process $x_{si,t+1}^{IB}=x_{si,t}^{IB}+\omega_{si}^{IB}x_{si,t}^{IB}\frac{\partial \pi_{si,t}^{IB}}{\partial x_{si,t}^{IB}},i=1,2,3,4$ of the output decisions of the PM chain si, and by setting $x_{si,t-1}^{IB}=q_{si,t}^{IB},i=1,2,3,4$, the discrete dynamic system model of the chain-to-chain game of the closed-loop supply chain system of CBEC under the IB scenario can be expressed as:

(34) $$\begin{array}{l} x_{s1,t+1}^{IB}=x_{s1,t}^{IB}+\omega_{s1}^{IB}x_{s1,t}^{IB}((u-2x_{s1,t}^{IB}-\beta (x_{s2,t}^{IB}+x_{s3,t}^{IB}+x_{s4,t}^{IB}))(1+\varphi_{CE})(1+\chi_{c})-2\gamma_{1}x_{s1,t}^{IB}+\gamma_{2}q_{s1,t}^{IB}-\gamma_{4})\\ x_{s2,t+1}^{IB}=x_{s2,t}^{IB}+\omega_{s2}^{IB}x_{s2,t}^{IB}((u-2x_{s2,t}^{IB}-\beta (x_{s1,t}^{IB}+x_{s3,t}^{IB}+x_{s4,t}^{IB}))(1+\chi_{e})\Delta_{s2}-2\gamma_{6}x_{s2,t}^{IB}+\gamma_{7}q_{s2,t}^{IB}-\gamma_{9})\\ x_{s3,t+1}^{IB}=x_{s3,t}^{IB}+\omega_{s3}^{IB}x_{s3,t}^{IB}((u-2x_{s3,t}^{IB}-\beta (x_{s1,t}^{IB}+x_{s2,t}^{IB}+x_{s4,t}^{IB}))(1+\varphi_{LE})(1+\chi_{e})\Delta_{s3}-2\gamma_{11}x_{s3,t}^{IB}+\gamma_{12}q_{s3,t}^{IB}-\gamma_{14})\\ x_{s4,t+1}^{IB}=x_{s4,t}^{IB}+\omega_{s4}^{IB}x_{s4,t}^{IB}((u-2x_{s4,t}^{IB}-\beta (x_{s1,t}^{IB}+x_{s2,t}^{IB}+x_{s3,t}^{IB}))(1+\varphi_{LE})(1+\chi_{e})\Delta_{s4}-2\gamma_{16}x_{s4,t}^{IB}+\gamma_{17}q_{s4,t}^{IB}-\gamma_{19})\\ q_{s1,t+1}^{IB}=x_{s1,t}^{IB}\\ q_{s2,t+1}^{IB}=x_{s2,t}^{IB}\\ q_{s3,t+1}^{IB}=x_{s3,t}^{IB}\\ q_{s4,t+1}^{IB}=x_{s4,t}^{IB} \end{array}$$

### 3.4. Information Sharing and Direct-Mail (ID)

#### 3.4.1. Inverse Demand Function of PM Chain

As the change in logistics mode does not affect the functional relationship between market prices and the output of products in each PM chain, the inverse demand functions of each PM chain under the ID scenario are the same as those under the IB scenario; that is, $P_{si,t}^{ID}=P_{si,t}^{IB},i=1,2,3,4$.

#### 3.4.2. The Cost for the Company Regarding Product $P^{*}$

Similarly, the information-sharing strategy only affects the estimation of product prices by the overseas manufacturer $a_{2}$, while the logistics mode influences the changes in the production output structure of the companies. Therefore, under the ID scenario, the composition of the production and operational costs for each company remains the same as under the ND scenario; that is, $C_{\varepsilon si,t}^{ID}=C_{\varepsilon si,t}^{ND},i=1,2,3,4$.

#### 3.4.3. The Transaction Cost for the Links Regarding $P^{*}$

As the production structure of the companies is the same as that under the ND scenario, the composition of the transaction costs under the ID scenario is identical to that under the ND scenario; that is, $C_{b_{m}si,t}^{ID}=C_{b_{m}si,t}^{ND},i=1,2,3,4$.

#### 3.4.4. Discrete Dynamic Model of the System

Considering the above, the composition of the total costs for each PM chain under the ID scenario is identical to that under the ND scenario; that is, $C_{si,t}^{ID}=C_{si,t}^{ND},i=1,2,3,4$.

Under the ID scenario, the profits of the four PM chains are as follows:

(35) $$\left\{ \begin{array}{l} \pi_{si,t}^{ID}=P_{si,t}^{ID}x_{si,t}^{ID}-C_{si,t}^{ID},i=1,3,4\\ \pi_{s2,t}^{ID}=P_{s2,t}^{ID}\Delta_{s2}x_{s2,t}^{ID}-C_{s2,t}^{ID} \end{array}\right.$$

where the concrete expression of Formula (35) is detailed in Appendix C.

The profit of the PM chain si is the difference between its revenue and cost, so under the ID scenario, the profits of four PM chains are as follows:

(36) $$\begin{array}{l} x_{s1,t+1}^{ID}=x_{s1,t}^{ID}+\omega_{s1}^{ID}x_{s1,t}^{ID}((u-2x_{s1,t}^{ID}-\beta (x_{s2,t}^{ID}+x_{s3,t}^{ID}+x_{s4,t}^{ID}))(1+\varphi_{CE})(1+\chi_{c})-2\gamma_{1}x_{s1,t}^{ID}+\gamma_{2}q_{s1,t}^{ID}-\gamma_{4})\\ x_{s2,t+1}^{ID}=x_{s2,t}^{ID}+\omega_{s2}^{ID}x_{s2,t}^{ID}((u-2x_{s2,t}^{ID}-\beta (x_{s1,t}^{ID}+x_{s3,t}^{ID}+x_{s4,t}^{ID}))(1+\chi_{e})\Delta_{s2}-2\gamma_{6}x_{s2,t}^{ID}+\gamma_{7}q_{s2,t}^{ID}-\gamma_{9})\\ x_{s3,t+1}^{ID}=x_{s3,t}^{ID}+\omega_{s3}^{ID}x_{s3,t}^{ID}((u-2x_{s3,t}^{ID}-\beta (x_{s1,t}^{ID}+x_{s2,t}^{ID}+x_{s4,t}^{ID}))(1+\varphi_{LE})(1+\chi_{e})-2\gamma_{21}x_{s3,t}^{ID}+\gamma_{22}q_{s3,t}^{ID}-\gamma_{24})\\ x_{s4,t+1}^{ID}=x_{s4,t}^{ID}+\omega_{s4}^{ID}x_{s4,t}^{ID}((u-2x_{s4,t}^{ID}-\beta (x_{s1,t}^{ID}+x_{s2,t}^{ID}+x_{s3,t}^{ID}))(1+\varphi_{LE})(1+\chi_{e})-2\gamma_{26}x_{s4,t}^{ID}+\gamma_{22}q_{s4,t}^{ID}-\gamma_{27})\\ q_{s1,t+1}^{ID}=x_{s1,t}^{ID}\\ q_{s2,t+1}^{ID}=x_{s2,t}^{ID}\\ q_{s3,t+1}^{ID}=x_{s3,t}^{ID}\\ q_{s4,t+1}^{ID}=x_{s4,t}^{ID} \end{array}$$

## 4. Nash Equilibrium and Stability Conditions

In the discrete dynamical systems (18), (31), (34), and (35), 16 equilibrium solutions can be obtained by setting $x_{si,t+1}^{\sigma }=x_{si,t}^{\sigma }(\sigma =NB,ND,IB,ID;i=1,2,3,4)$ for the NB, ND, IB, and ID scenarios. Only at the equilibrium solution $E^{\sigma^{*}}(\sigma =NB,ND,IB,ID)$, zero output levels do not exist, which means that no player in the game can independently take actions to increase revenue. Therefore, $E^{\sigma^{*}}(\sigma =NB,ND,IB,ID)$ is the unique Nash equilibrium point of the system under the four scenarios.

The stability analysis of the Nash equilibrium points for the system under the NB, ND, IB, and ID scenarios is as follows:

The Jacobian matrix of the discrete dynamic systems (18), (31), (34), and (35) is as follows:

(37) $$J^{\sigma }=\left(\begin{array}{llllllll} J_{11}^{\sigma } & J_{12}^{\sigma } & J_{13}^{\sigma } & J_{14}^{\sigma } & J_{15}^{\sigma } & 0 & 0 & 0\\ J_{21}^{\sigma } & J_{22}^{\sigma } & J_{23}^{\sigma } & J_{24}^{\sigma } & 0 & J_{26}^{\sigma } & 0 & 0\\ J_{31}^{\sigma } & J_{32}^{\sigma } & J_{33}^{\sigma } & J_{34}^{\sigma } & 0 & 0 & J_{37}^{\sigma } & 0\\ J_{41}^{\sigma } & J_{42}^{\sigma } & J_{43}^{\sigma } & J_{44}^{\sigma } & 0 & 0 & 0 & J_{48}^{\sigma }\\ 1 & 0 & 0 & 0 & 0 & 0 & 0 & 0\\ 0 & 1 & 0 & 0 & 0 & 0 & 0 & 0\\ 0 & 0 & 1 & 0 & 0 & 0 & 0 & 0\\ 0 & 0 & 0 & 1 & 0 & 0 & 0 & 0 \end{array}\right),\sigma =NB,ND,IB,ID$$

where,

$$J_{11}^{\sigma }=\left\{ \begin{array}{l} 1+\omega_{s1}^{\sigma }((E(u_{x})-4x_{s1,t}^{\sigma }-\beta (x_{s2,t}^{\sigma }+x_{s3,t}^{\sigma }+x_{s4,t}^{\sigma }))(1+\varphi_{CE})(1+\chi_{c})-4\gamma_{1}x_{s1,t}^{\sigma }+\gamma_{2}q_{s1,t}^{\sigma }-\gamma_{4}),\sigma =NB,ND\\ 1+\omega_{s1}^{\sigma }((u-4x_{s1,t}^{\sigma }-\beta (x_{s2,t}^{\sigma }+x_{s3,t}^{\sigma }+x_{s4,t}^{\sigma }))(1+\varphi_{CE})(1+\chi_{c})-4\gamma_{1}x_{s1,t}^{\sigma }+\gamma_{2}q_{s1,t}^{\sigma }-\gamma_{4}),\sigma =IB,ID \end{array}\right.,$$

$$J_{22}^{\sigma }=1+\omega_{s2}^{\sigma }((u-4x_{s2,t}^{\sigma }-\beta (x_{s1,t}^{\sigma }+x_{s3,t}^{\sigma }+x_{s4,t}^{\sigma }))(1+\chi_{e})\Delta_{s2}-4\gamma_{6}x_{s2,t}^{\sigma }+\gamma_{7}q_{s2,t}^{\sigma }-\gamma_{9}),\sigma =NB,ND,IB,ID,$$

$$J_{33}^{NB}=1+\omega_{s3}^{NB}((E(u_{x})-4x_{s3,t}^{NB}-\beta (x_{s1,t}^{NB}+x_{s2,t}^{NB}+x_{s4,t}^{NB}))(1+\varphi_{LE})(1+\chi_{e})\Delta_{s3}-4\gamma_{11}x_{s3,t}^{NB}+\gamma_{12}q_{s3,t}^{NB}-\gamma_{14}),$$

$$J_{33}^{ND}=1+\omega_{s3}^{ND}((E(u_{x})-4x_{s3,t}^{ND}-\beta (x_{s1,t}^{ND}+x_{s2,t}^{ND}+x_{s4,t}^{ND}))(1+\varphi_{LE})(1+\chi_{e})-4\gamma_{21}x_{s3,t}^{ND}+\gamma_{22}q_{s3,t}^{ND}-\gamma_{24}),$$

$$J_{33}^{IB}=1+\omega_{s3}^{IB}((u-4x_{s3,t}^{IB}-\beta (x_{s1,t}^{IB}+x_{s2,t}^{IB}+x_{s4,t}^{IB}))(1+\varphi_{LE})(1+\chi_{e})\Delta_{s3}-4\gamma_{11}x_{s3,t}^{IB}+\gamma_{12}q_{s3,t}^{IB}-\gamma_{14}),$$

$$J_{33}^{ID}=1+\omega_{s3,t}^{ID}((u-4x_{s3,t}^{ID}-\beta (x_{s1,t}^{ID}+x_{s2,t}^{ID}+x_{s4,t}^{ID}))(1+\varphi_{LE})(1+\chi_{e})-4\gamma_{21}x_{s3,t}^{ID}+\gamma_{22}q_{s3,t}^{ID}-\gamma_{24}),$$

$$J_{44}^{NB}=1+\omega_{s4}^{NB}((E(u_{x})-4x_{s4,t}^{NB}-\beta (x_{s1,t}^{NB}+x_{s2,t}^{NB}+x_{s3,t}^{NB}))(1+\varphi_{LE})(1+\chi_{e})\Delta_{s4}-4\gamma_{16}x_{s4,t}^{NB}+\gamma_{17}q_{s4,t}^{NB}-\gamma_{19}),$$

$$J_{44}^{ND}=1+\omega_{s4}^{ND}((E(u_{x})-4x_{s4,t}^{ND}-\beta (x_{s1,t}^{ND}+x_{s2,t}^{ND}+x_{s3,t}^{ND}))(1+\varphi_{LE})(1+\chi_{e})-4\gamma_{26}x_{s4,t}^{ND}+\gamma_{22}q_{s4,t}^{ND}-\gamma_{27}),$$

$$J_{44}^{IB}=1+\omega_{s4}^{IB}((u-4x_{s4,t}^{IB}-\beta (x_{s1,t}^{IB}+x_{s2,t}^{IB}+x_{s3,t}^{IB}))(1+\varphi_{LE})(1+\chi_{e})\Delta_{s4}-4\gamma_{16}x_{s4,t}^{IB}+\gamma_{17}q_{s4,t}^{IB}-\gamma_{19}),$$

$$J_{44}^{ID}=1+\omega_{s4}^{ID}((u-4x_{s4,t}^{ID}-\beta (x_{s1,t}^{ID}+x_{s2,t}^{ID}+x_{s3,t}^{ID}))(1+\varphi_{LE})(1+\chi_{e})-4\gamma_{26}x_{s4,t}^{ID}+\gamma_{22}q_{s4,t}^{ID}-\gamma_{27}),$$

$$J_{12}^{\sigma }=J_{13}^{\sigma }=J_{14}^{\sigma }=-\beta (1+\varphi_{CE})(1+\chi_{c})\omega_{s1}^{\sigma }x_{s1,t}^{\sigma },\sigma =NB,ND,IB,ID,J_{15}^{\sigma }=\gamma_{2}\omega_{s1}^{\sigma }x_{s1,t}^{\sigma },\sigma =NB,ND,IB,ID,$$

$$J_{21}^{\sigma }=J_{23}^{\sigma }=J_{24}^{\sigma }=-\beta (1+\chi_{e})\Delta_{s2}\omega_{s2}^{\sigma }x_{s2,t}^{\sigma },\sigma =NB,ND,IB,ID,J_{26}^{\sigma }=\gamma_{7}\omega_{s2}^{\sigma }x_{s2,t}^{\sigma },\sigma =NB,ND,IB,ID,$$

$$J_{31}^{\sigma }=J_{32}^{\sigma }=J_{34}^{\sigma }=\left\{ \begin{array}{l} -\beta (1+\varphi_{LE})(1+\chi_{e})\Delta_{s3}\omega_{s3}^{\sigma }x_{s3,t}^{\sigma },\sigma =NB,IB\\ -\beta (1+\varphi_{LE})(1+\chi_{e})\omega_{s3}^{\sigma }x_{s3,t}^{\sigma },\sigma =ND,ID \end{array}\right.,J_{37}^{\sigma }=\left\{ \begin{array}{l} \gamma_{12}\omega_{s3}^{\sigma }x_{s3,t}^{\sigma },\sigma =NB,IB\\ \gamma_{22}\omega_{s3}^{\sigma }x_{s3,t}^{\sigma },\sigma =ND,ID \end{array}\right.,$$

$$J_{41}^{\sigma }=J_{42}^{\sigma }=J_{43}^{\sigma }=\left\{ \begin{array}{l} -\beta (1+\varphi_{LE})(1+\chi_{e})\Delta_{s4}\omega_{s4}^{\sigma }x_{s4,t}^{\sigma },\sigma =NB,IB\\ -\beta (1+\varphi_{LE})(1+\chi_{e})\omega_{s4}^{\sigma }x_{s4,t}^{\sigma },\sigma =ND,ID \end{array}\right.,J_{48}^{\sigma }=\left\{ \begin{array}{l} \gamma_{17}\omega_{s4}^{\sigma }x_{s4,t}^{\sigma },\sigma =NB,IB\\ \gamma_{22}\omega_{s4}^{\sigma }x_{s4,t}^{\sigma },\sigma =ND,ID \end{array}\right..$$

The characteristic polynomial of the Jacobian matrix (37) is obtained as follows:

(38) $$F^{\sigma }(\lambda )=\lambda^{8}+B_{7}^{\sigma }\lambda^{7}+B_{6}^{\sigma }\lambda^{6}+B_{5}^{\sigma }\lambda^{5}+B_{4}^{\sigma }\lambda^{4}+B_{3}^{\sigma }\lambda^{3}+B_{2}^{\sigma }\lambda^{2}+B_{1}^{\sigma }\lambda +B_{0}^{\sigma }$$

where the concrete expressions of $B_{0}^{\sigma }$–$B_{7}^{\sigma }$ are detailed in Appendix D.

According to the Jury criterion [31], the conditions for the asymptotic stability of the discrete dynamic systems (18), (31), (34), and (36) are as follows:

(39) $$\{\begin{array}{l} F^{\sigma }(1)=1+B_{7}^{\sigma }+B_{6}^{\sigma }+B_{5}^{\sigma }+B_{4}^{\sigma }+B_{3}^{\sigma }+B_{2}^{\sigma }+B_{1}^{\sigma }+B_{0}^{\sigma }>0\\ F^{\sigma }(-1)=1-B_{7}^{\sigma }+B_{6}^{\sigma }-B_{5}^{\sigma }+B_{4}^{\sigma }-B_{3}^{\sigma }+B_{2}^{\sigma }-B_{1}^{\sigma }+B_{0}^{\sigma }>0\\ \left|B_{0}^{\sigma }\right|<1\\ \left|D_{0}^{\sigma }\right|>\left|D_{7}^{\sigma }\right|\\ \left|G_{0}^{\sigma }\right|>\left|G_{6}^{\sigma }\right|\\ \left|H_{0}^{\sigma }\right|>\left|H_{5}^{\sigma }\right|\\ \begin{array}{l} \left|Z_{0}^{\sigma }\right|>\left|Z_{4}^{\sigma }\right|\\ \left|Q_{0}^{\sigma }\right|>\left|Q_{3}^{\sigma }\right|\\ \left|O_{0}^{\sigma }\right|>\left|O_{2}^{\sigma }\right| \end{array} \end{array}$$

where, $D_{0}^{\sigma }=\left|\begin{array}{cc} B_{0}^{\sigma } & 1\\ 1 & B_{0}^{\sigma } \end{array}\right|$, $D_{1}^{\sigma }=\left|\begin{array}{cc} B_{0}^{\sigma } & B_{7}^{\sigma }\\ 1 & B_{1}^{\sigma } \end{array}\right|$, $D_{2}^{\sigma }=\left|\begin{array}{cc} B_{0}^{\sigma } & B_{6}^{\sigma }\\ 1 & B_{2}^{\sigma } \end{array}\right|$, $D_{3}^{\sigma }=\left|\begin{array}{cc} B_{0}^{\sigma } & B_{5}^{\sigma }\\ 1 & B_{3}^{\sigma } \end{array}\right|$, $D_{4}^{\sigma }=\left|\begin{array}{cc} B_{0}^{\sigma } & B_{4}^{\sigma }\\ 1 & B_{4}^{\sigma } \end{array}\right|$, $D_{5}^{\sigma }=\left|\begin{array}{cc} B_{0}^{\sigma } & B_{3}^{\sigma }\\ 1 & B_{5}^{\sigma } \end{array}\right|$,$D_{6}^{\sigma }=\left|\begin{array}{cc} B_{0}^{\sigma } & B_{2}^{\sigma }\\ 1 & B_{6}^{\sigma } \end{array}\right|$, $D_{7}^{\sigma }=\left|\begin{array}{cc} B_{0}^{\sigma } & B_{1}^{\sigma }\\ 1 & B_{7}^{\sigma } \end{array}\right|$, $G_{0}^{\sigma }=\left|\begin{array}{cc} D_{0}^{\sigma } & D_{7}^{\sigma }\\ D_{7}^{\sigma } & D_{0}^{\sigma } \end{array}\right|$, $G_{1}^{\sigma }=\left|\begin{array}{cc} D_{0}^{\sigma } & D_{6}^{\sigma }\\ D_{7}^{\sigma } & D_{1}^{\sigma } \end{array}\right|$, $G_{2}^{\sigma }=\left|\begin{array}{cc} D_{0}^{\sigma } & D_{5}^{\sigma }\\ D_{7}^{\sigma } & D_{2}^{\sigma } \end{array}\right|$, $G_{3}^{\sigma }=\left|\begin{array}{cc} D_{0}^{\sigma } & D_{4}^{\sigma }\\ D_{7}^{\sigma } & D_{3}^{\sigma } \end{array}\right|$, $G_{4}^{\sigma }=\left|\begin{array}{cc} D_{0}^{\sigma } & D_{3}^{\sigma }\\ D_{7}^{\sigma } & D_{4}^{\sigma } \end{array}\right|$, $G_{5}^{\sigma }=\left|\begin{array}{cc} D_{0}^{\sigma } & D_{2}^{\sigma }\\ D_{7}^{\sigma } & D_{5}^{\sigma } \end{array}\right|$, $G_{6}^{\sigma }=\left|\begin{array}{cc} D_{0}^{\sigma } & D_{1}^{\sigma }\\ D_{7}^{\sigma } & D_{6}^{\sigma } \end{array}\right|$, $H_{0}^{\sigma }=\left|\begin{array}{cc} G_{0}^{\sigma } & G_{6}^{\sigma }\\ G_{6}^{\sigma } & G_{0}^{\sigma } \end{array}\right|$, $H_{1}^{\sigma }=\left|\begin{array}{cc} G_{0}^{\sigma } & G_{5}^{\sigma }\\ G_{6}^{\sigma } & G_{1}^{\sigma } \end{array}\right|$, $H_{2}^{\sigma }=\left|\begin{array}{cc} G_{0}^{\sigma } & G_{4}^{\sigma }\\ G_{6}^{\sigma } & G_{2}^{\sigma } \end{array}\right|$, $H_{3}^{\sigma }=\left|\begin{array}{cc} G_{0}^{\sigma } & G_{3}^{\sigma }\\ G_{6}^{\sigma } & G_{3}^{\sigma } \end{array}\right|$, $H_{4}^{\sigma }=\left|\begin{array}{cc} G_{0}^{\sigma } & G_{2}^{\sigma }\\ G_{6}^{\sigma } & G_{4}^{\sigma } \end{array}\right|$, $H_{5}^{\sigma }=\left|\begin{array}{cc} G_{0}^{\sigma } & G_{1}^{\sigma }\\ G_{6}^{\sigma } & G_{5}^{\sigma } \end{array}\right|$, $Z_{0}^{\sigma }=\left|\begin{array}{cc} H_{0}^{\sigma } & H_{5}^{\sigma }\\ H_{5}^{\sigma } & H_{0}^{\sigma } \end{array}\right|$, $Z_{1}^{\sigma }=\left|\begin{array}{cc} H_{0}^{\sigma } & H_{4}^{\sigma }\\ H_{5}^{\sigma } & H_{1}^{\sigma } \end{array}\right|$, $Z_{2}^{\sigma }=\left|\begin{array}{cc} H_{0}^{\sigma } & H_{3}^{\sigma }\\ H_{5}^{\sigma } & H_{2}^{\sigma } \end{array}\right|$, $Z_{3}^{\sigma }=\left|\begin{array}{cc} H_{0}^{\sigma } & H_{2}^{\sigma }\\ H_{5}^{\sigma } & H_{3}^{\sigma } \end{array}\right|$, $Z_{4}^{\sigma }=\left|\begin{array}{cc} H_{0}^{\sigma } & H_{1}^{\sigma }\\ H_{5}^{\sigma } & H_{4}^{\sigma } \end{array}\right|$, $Q_{0}^{\sigma }=\left|\begin{array}{cc} Z_{0}^{\sigma } & Z_{4}^{\sigma }\\ Z_{4}^{\sigma } & Z_{0}^{\sigma } \end{array}\right|$, $Q_{1}^{\sigma }=\left|\begin{array}{cc} Z_{0}^{\sigma } & Z_{3}^{\sigma }\\ Z_{4}^{\sigma } & Z_{1}^{\sigma } \end{array}\right|$, $Q_{2}^{\sigma }=\left|\begin{array}{cc} Z_{0}^{\sigma } & Z_{2}^{\sigma }\\ Z_{4}^{\sigma } & Z_{2}^{\sigma } \end{array}\right|$, $Q_{3}^{\sigma }=\left|\begin{array}{cc} Z_{0}^{\sigma } & Z_{1}^{\sigma }\\ Z_{4}^{\sigma } & Z_{3}^{\sigma } \end{array}\right|$, $O_{0}^{\sigma }=\left|\begin{array}{cc} Q_{0}^{\sigma } & Q_{3}^{\sigma }\\ Q_{3}^{\sigma } & Q_{0}^{\sigma } \end{array}\right|$, $O_{1}^{\sigma }=\left|\begin{array}{cc} Q_{0}^{\sigma } & Q_{2}^{\sigma }\\ Q_{3}^{\sigma } & Q_{1}^{\sigma } \end{array}\right|$, $O_{2}^{\sigma }=\left|\begin{array}{cc} Q_{0}^{\sigma } & Q_{1}^{\sigma }\\ Q_{3}^{\sigma } & Q_{2}^{\sigma } \end{array}\right|$.

The region formed by expression (39)with respect to $\omega_{si}^{\sigma }$ is referred to as the system stable region. When the values of $\omega_{si}^{\sigma }$ are within these stable regions, the output decisions of the four PM chains gradually stabilize to the Nash equilibrium point $E^{\sigma^{*}}$ in competition.

## 5. Numerical Simulation Analysis

In view of the complexity of the closed-loop supply chain system of CBEC considered in this study, this section builds on the above theoretical analysis by employing MATLAB (R2018a) and EFchaos software (version 1.00) for numerical simulations.

Using the closed-loop supply chain system of CBEC for wireless telephones as an example, the parameters are set as follows:

(1) Product pricing and commission rates: On the JD.com platform in China, the official price u of an imported Panasonic cordless telephone is approximately USD 70, while the lowest price is around USD 10. As the average price $\bar{u}$ is difficult to obtain, this study calculates the arithmetic mean price based on publicly available sales records for this product on e-commerce platforms, resulting in an average price $\bar{u}$ of about USD 16.5. Furthermore, according to the commission table for the JD.com platform, the commission rates for goods mostly range from 1% to 10% [32]. In this study, we assume that both overseas and domestic e-commerce platforms have a commission rate ($\varphi_{LE}$,$\varphi_{CE}$) of 10%.

(2) Price distribution: According to the literature [33], when the average price $\bar{u}$ of a product reaches a certain level, a small number of items are priced high, while the majority are priced low; that is, the product prices follow a negative exponential distribution with respect to the reciprocal of the average price. Therefore, we assume that the prices of wireless telephones in this system also follow such an inverse exponential distribution; namely, $u_{x}\sim E(\frac{1}{\bar{u}})$.

(3) Tax rates: Based on data from the General Administration of Customs of the People’s Republic of China [34], the VAT rate for wireless telephones is 13%, the consumption tax is 0%, and the general import tariff rate is 20%, resulting in an overall import comprehensive tax rate of 33%. Regarding the CBEC comprehensive tax, wireless telephones also have a VAT rate of 13%, with the consumption tax remaining at 0%. As VAT and consumption tax for CBEC transactions are 70% of those for regular trade imports, the consumer CBEC comprehensive tax rate is calculated, accordingly, as $\chi_{e}=\frac{0+13\%}{1-0\%}\times 70\%=9.1\%$ [35].

(4) Marginal costs and substitutability: As the marginal costs of companies and links, as well as the substitutability between different channels, are difficult to measure directly, we refer to simulation parameters from the related literature on inter-chain competition economic models [27,28]. The parameter settings are as follows: in terms of marginal cost, we assume that the marginal production cost of the overseas raw material supplier $a_{1}$ for new products is $r_{a_{1}}=0.1$, while that for re-manufactured products is $r_{a_{1}}^{-}=0.05$; the marginal production cost of the overseas manufacturer $a_{2}$ is $r_{a_{2}}=0.1$; the marginal operating costs of the overseas CBEC platform CE and the domestic CBEC platform LE are $r_{\mathrm{CE}}=0.1$ and $r_{\mathrm{LE}}=0.1$, respectively; and the marginal storage cost of bonded warehouses BW is $r_{\mathrm{BW}}=0.1$. Considering that e-commerce platforms attach importance to brands and customer experience, they tend to provide better, more reliable logistics services. Therefore, we assume that the marginal operating cost of the domestic platforms’ self-built logistic company PL is $r_{\mathrm{PL}}=0.2$, while those of the overseas international logistic company IL and domestic third-party logistics company TL are $r_{\mathrm{IL}}=0.1$ and $r_{\mathrm{TL}}=0.1$, respectively. In addition, cross-border transactions may incur higher search and negotiation costs due to information asymmetry. Therefore, we assume that the marginal transaction costs of cross-border links $b_{4}$, $b_{5}$, and $b_{9}$ are 0.2, while the marginal transaction costs on the remaining links are 0.1. We assume that the substitutability rate among the four channels (PM chains s1–s4) is β=0.4 and that the overseas raw material supplier $a_{1}$ and overseas manufacturer $a_{2}$ need to provide two units of materials for one unit of the final product; thus, $\rho_{a1}=\rho_{a2}=2$.

(5) Other parameter assumptions: All parameters in this study aim to simulate dynamic behaviors under specific market conditions and, as such, do not encompass all market scenarios. These parameter choices provide a clear framework for simulating the closed-loop supply chain system of CBEC, facilitating the exploration of output decision evolution and the potential impacts of policy adjustments. For data that are difficult to estimate precisely, such as the recycling rate of wireless telephones and the transaction ratios in bonded warehouses, reasonable assumptions were made: the recycling rate of wireless telephone products in the market MR is υ=50% and the proportion of recycled products that can be re-manufactured is η=50%; furthermore, under the bonded warehouse mode, the actual transaction ratios of wireless telephone products in the PM chains s2, s3, and s4 are $\Delta_{s2}=60\%$, $\Delta_{s3}=60\%$, and $\Delta_{s4}=20\%$, respectively. These transaction ratios $\Delta_{s3}$ and $\Delta_{s4}$ are set to different values to ensure that clear distinctions can be observed in the graphical representations.

### 5.1. The System Stability

The output adjustment speed $\omega_{si}^{\sigma }$ for the PM chain is a key parameter reflecting decision-making characteristics. An aggressive PM chain would opt for a larger output adjustment speed $\omega_{si}^{\sigma }$ to rapidly increase profits, while a cautious PM chain would choose a smaller output adjustment speed $\omega_{si}^{\sigma }$ to mitigate risks. To demonstrate the impact of the PM chain’s output adjustment speed $\omega_{si}^{\sigma }$ on the system’s output decisions, in this paper, we fix the $\omega_{si}^{\sigma }$ at 0.015.

**Figure 2:**
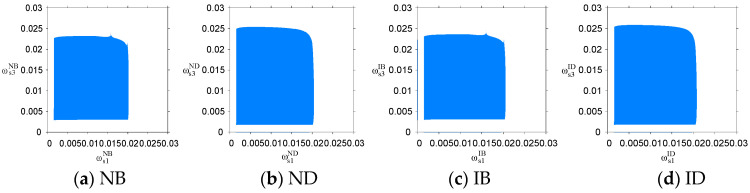
Stable regions of the system with respect to $\omega_{s1}^{\sigma }$ and $\omega_{s3}^{\sigma }$ under the (**a**) NB, (**b**) ND, (**c**) IB, and (**d**) ID scenarios.

The output adjustment speeds $\omega_{s2}^{\sigma }$ and $\omega_{s4}^{\sigma }$ of PM chains s2 and s4 were fixed, and the stable regions of the system’s output decisions with respect to $\omega_{s1}^{\sigma }$ and $\omega_{s3}^{\sigma }$ under the four scenarios are shown in Figure 2. When the output adjustment speeds $\omega_{s1}^{\sigma }$ and $\omega_{s3}^{\sigma }$ were within respective stable regions under four scenarios, the output decisions of the cross-border supply chain system remained at the Nash equilibrium point $E^{\sigma^{*}}$; otherwise, the system’s output decisions no longer remained stable. Therefore, under the NB, ND, IB, and ID scenarios, the output adjustment speed of PM chain s1 in each period should not exceed 0.02, while the output adjustment speeds of PM chain s3 should not exceed 0.023, 0.0245, 0.023, and 0.0245, respectively. A comparison of Figure 2a,b with Figure 2c,d indicates that the stable regions of the closed-loop supply chain system of CBEC with respect to $\omega_{s1}^{\sigma }$ and $\omega_{s3}^{\sigma }$ are larger under the direct-mail mode. This indicates that, compared with the bonded warehouse mode, the direct-mail mode (scenarios ND and ID) can enhance the stability of the system’s output decisions. The advantage of the direct-mail mode in expanding the stability region may be attributed to its faster response time and lower inventory risk.

**Figure 3:**
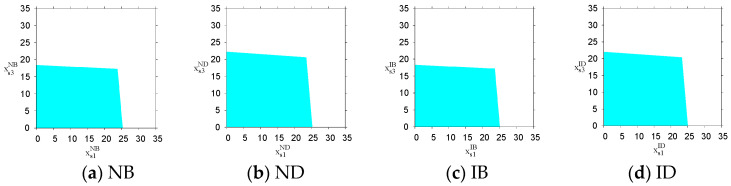
Stable regions of the system with respect to the initial output levels of s1 and s3 under the (**a**) NB, (**b**) ND, (**c**) IB, and (**d**) ID scenarios.

The initial output levels of the PM chain significantly influence the stability of the cross-border supply chain system, and not all initial output levels can lead the system to a stable state. Figure 3 shows the stable regions with respect to the initial output levels of s1 and s3 under the four scenarios with the cyan area. When the initial output levels of PM chains s1 and s3 are within the stable regions shown in Figure 3, the system’s output decisions ultimately converge to the Nash equilibrium point $E^{\sigma^{*}}$. Otherwise, the system’s output decisions enter an unstable state. Figure 3 indicates that, under the bonded warehouse mode (NB and IB) and the direct-mail mode (ND and ID), the initial output levels of PM chains s1 and s3 should be controlled below 23 and 17 and 23 and 20, respectively. Therefore, the system’s stable regions regarding the initial output levels are larger under the direct-mail mode when compared to the bonded warehouse mode. Under the bonded warehouse mode, PM chains s1 and s3 should be more cautious in selecting their initial output levels in order to ensure that they do not exceed the specified thresholds; otherwise, regardless of the output adjustment speed, the system’s output decisions will never reach a stable state. The smaller region of stability under the bonded warehouse mode reflects its limitations in inventory management and output planning, as higher initial output levels may lead to instability due to the lag effect of inventory accumulation. In contrast, the direct-mail mode demonstrates a larger stable range for initial output by reducing inventory backlogs and directly responding to market demand, making it particularly suitable for highly volatile markets.

Integrating Figure 2 and Figure 3, the combination of the direct-mail mode and information-sharing strategy (ID) demonstrated the best stability, in terms of both adjustment speed and initial output. This indicates that the direct-mail mode offers significant advantages in rapidly adapting to market changes, while the information-sharing strategy further amplifies its region of stability, providing theoretical support for supply chain management. For the governments of importing countries and supply chain participants, this finding suggests that prioritizing a combination of the direct-mail mode and information-sharing strategy when formulating logistics models and collaborative strategies can enhance the overall dynamic stability of the supply chain.

**Figure 4:**
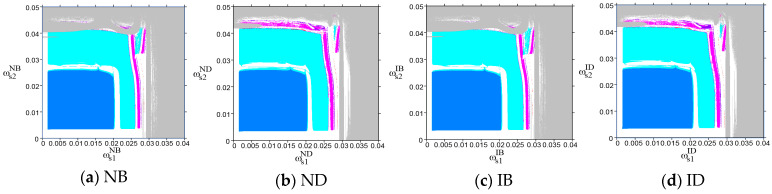
Two-dimensional bifurcation diagrams of the system with respect to $\omega_{s1}^{\sigma }$ and $\omega_{s2}^{\sigma }$ under the (**a**) NB, (**b**) ND, (**c**) IB, and (**d**) ID scenarios.

To further explore the dynamic evolution of the system when the output adjustment speed $\omega_{si}^{\sigma }$ is outside the stable regions, Figure 4 shows the two-dimensional bifurcation diagrams of the system’s output decisions as the output adjustment speeds $\omega_{s1}^{\sigma }$ and $\omega_{s2}^{\sigma }$ vary under the four scenarios. The blue, cyan, magenta, purple, and red regions represent the stable state and the two-, four-, six-, and eight-cycle states of the system’s output decisions, respectively, and the white and gray represent the chaotic state and divergence area. When the system is in a chaotic state, the output decisions exhibit randomness and volatility, while in a divergent state, the output decisions become unpredictable and uncontrollable.

Figure 4 shows that, from the perspective of the output adjustment speed $\omega_{s1}^{\sigma }$ of the PM chain s1, the system’s output decisions under the four scenarios lose stability at $\omega_{s1}^{\sigma }=0.02$ and enter a chaotic state characterized by random and fluctuating output decisions via a series of period-doubling bifurcations. Ultimately, the system transitions from a chaotic state to a divergent state where the output decisions become unpredictable. However, from the perspective of the output adjustment speed $\omega_{s2}^{\sigma }$ of the PM chain s2, the dynamic evolution of the system’s output decisions varies under different scenarios. Under the ND and ID scenarios, the system loses stability at $\omega_{s2}^{\sigma }=0.025$, then enters a chaotic state via a series of period-doubling bifurcations, and ultimately evolves to a divergent state. By contrast, under the NB and IB scenarios, the system directly enters a divergent state via a series of period-doubling bifurcations after losing stability. This indicates that the choice of logistics mode by the overseas manufacturer ($a_{2}$) significantly impacts the system’s dynamic evolution, particularly as the output adjustment speed $\omega_{s2}^{\sigma }$ changes. When employing the direct-mail logistics mode (ND and ID), output decisions initially exhibit fluctuations and disorder as $\omega_{s2}^{\sigma }$ increases, and as $\omega_{s2}^{\sigma }$ continues to rise, the output decisions become unpredictable and uncontrollable. In contrast, under the bonded warehouse logistics mode (NB and IB), an increase in $\omega_{s2}^{\sigma }$ causes the system to bypass the phase of fluctuations and disorder, directly entering a stage where the output decisions become unpredictable and uncontrollable. This highlights the importance of controlling the output adjustment speed $\omega_{s2}^{\sigma }$ carefully, particularly under the bonded warehouse mode. Specifically, to prevent the system from reaching an uncontrollable state, $\omega_{s2}^{\sigma }$ should be kept below 0.025.

**Figure 5:**
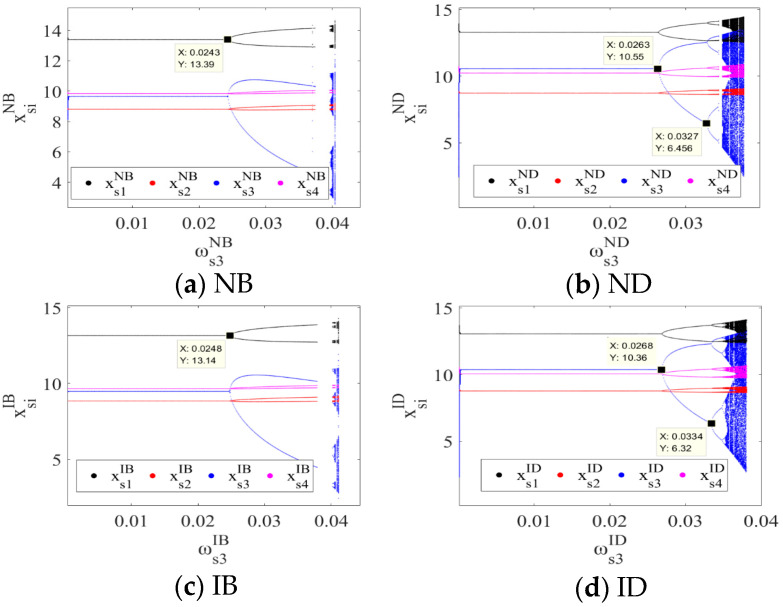
Bifurcation diagrams of the system with respect to $\omega_{s3}^{\sigma }$ under the (**a**) NB, (**b**) ND, (**c**) IB, and (**d**) ID scenarios.

Figure 5 shows the bifurcation diagrams of the system’s output decisions as the output adjustment speed $\omega_{s3}^{\sigma }$ of the PM chain s3 varies. Under the NB scenario, the system’s output decisions lose stability at $\omega_{s3}^{NB}=0.0243$, where the bifurcation occurs, and the system’s output decisions enter a two-cycle state. As $\omega_{s3}^{\sigma }$ further increases, the system’s output decisions enter a chaotic state. Under the ND scenario, the system loses stability at $\omega_{s3}^{ND}=0.0263$, then enters a two-cycle state, and finally evolves into a chaotic state. Similarly, under the IB and ID scenarios, the points at which the system loses stability are $\omega_{s3}^{IB}=0.0248$ and $\omega_{s3}^{ID}=0.0268$, respectively, and then the system enters a chaotic state via a series of period-doubling bifurcations. Therefore, under the four scenarios, an increase in $\omega_{s3}^{\sigma }$ causes the system to lose stability and ultimately leads to fluctuations and chaos in the output decisions of the closed-loop supply chain system of CBEC. The adjustment speed $\omega_{s3}^{\sigma }$ of the PM chain s3 should not exceed the corresponding threshold values mentioned above. In particular, under the NB and IB scenarios, when $0.0374<\omega_{s3}^{NB}<0.0396$ and $0.0379<\omega_{s3}^{IB}<0.0402$, the bifurcation diagram exhibits blank areas. This indicates that within these areas, the system’s output decisions become unpredictable and uncontrollable. Therefore, under the bonded warehouse mode (NB and IB), the output adjustment speed of the PM chain s3 should avoid values within $0.0374<\omega_{s3}^{NB}<0.0396$ and $0.0379<\omega_{s3}^{IB}<0.0402$ to ensure system stability and mitigate potential operational risks.

The comparison of the points at which the system loses stability (0.0243, 0.0263, 0.0248, and 0.0268) reveals that, relative to the bonded warehouse logistics mode (NB and IB), the direct-mail logistics mode (ND and ID) expands the stable range of the system’s decisions and enhances overall stability. Similarly, when comparing the non-information-sharing strategy (NB and ND) with the information-sharing strategy (IB and ID), the latter also increases the stable range of system decisions and further improves system stability. Among all four scenarios, the combination of information sharing and the direct-mail mode (ID) provides the maximum stability for the system’s output decision-making. To optimize stability, the domestic CBEC platform should share product market information with supply chain partners, promoting transparency and refining the supply chain decision-making process. At the same time, the overseas manufacturer should prioritize the direct-mail logistics mode to maintain flexibility in adjusting output levels, ensuring a balance between production and market demand.

**Figure 6:**
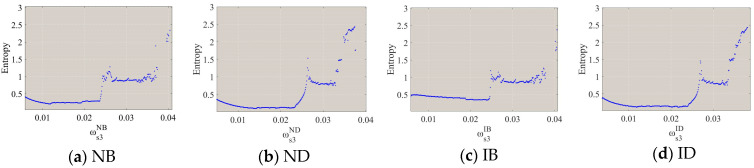
Entropy diagrams of the system with respect to $\omega_{s3}^{\sigma }$ under the (**a**) NB, (**b**) ND, (**c**) IB, and (**d**) ID scenarios.

Figure 6 shows the entropy of the system output decisions under four scenarios as the output adjustment speed $\omega_{s3}^{\sigma }$ of the PM chain s3 varies. In a stable state, the entropy value is relatively low. However, as the system transitions into bifurcation and chaotic states, the entropy value increases significantly, indicating a rise in the complexity and unpredictability of the system’s behavior.

**Figure 7:**
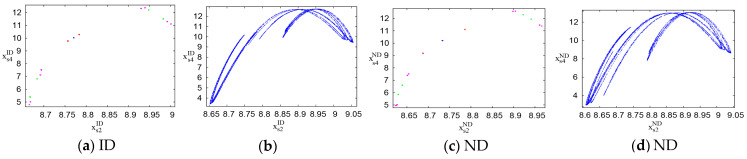
The attractors of the system under the (**a**,**b**) ID scenario with respect to different $\omega_{s4}^{IB}$; (**c**,**d**) ND scenario with respect to different $\omega_{s4}^{ND}$. (**a**) $\omega_{s4}^{IB}=0.015,0.027,0.033,0.0343$; (**b**) $\omega_{s4}^{IB}=0.037$; (**c**) $\omega_{s4}^{ND}=0.015,0.027,0.033,0.0343$; (**d**) $\omega_{s4}^{ND}=0.037$.

Figure 7 shows the attractors of the system’s output decisions as the output adjustment speed $\omega_{s4}^{\sigma }(\sigma =ID,ND)$ of the PM chain varies under the ID and ND scenarios. When the speeds $\omega_{s4}^{\sigma }(\sigma =ID,ND)$ are 0.015, 0.027, 0.033, and 0.0343, the stable point, 2-cycle, 4-cycle, and 8-cycle attractors of the output decisions under the ID and ND scenarios are marked by blue, red, green, and magenta dots, respectively, as shown in Figure 7a,c. Figure 7b,d show the chaotic attractors of the system under the ID and ND scenarios when the output adjustment speeds $\omega_{s4}^{\sigma }(\sigma =ID,ND)$ are 0.037, indicating that the system’s output decisions have entered a state of randomness and fluctuation. Consequently, as the output adjustment speeds $\omega_{s4}^{\sigma }(\sigma =ID,ND)$ increase, the attractors of the system’s output decisions gradually shift from a stable point to a chaotic attractor, with the system’s output decisions transitioning from a stable state to a state of fluctuation and disorder. This indicates that under the ID and ND scenarios, an excessively large output adjustment speed is detrimental to the stability of the system’s output decisions, and the system loses stability when the $\omega_{s4}^{\sigma }(\sigma =ID,ND)$ exceeds or equals 0.027. Therefore, under the ID and ND scenarios, PM chains should ensure that the output adjustment speeds remain below 0.027.

**Figure 8:**
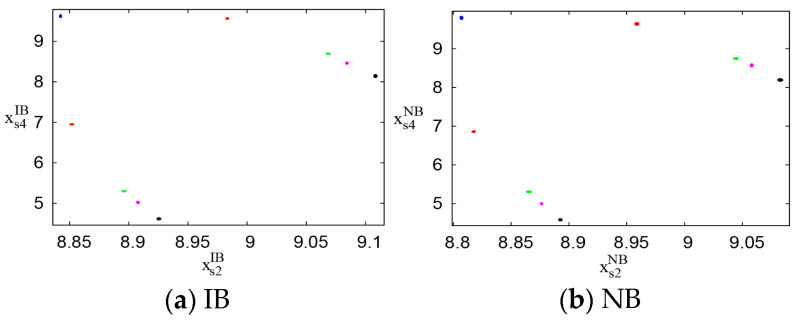
The attractors of the system under the (**a**) IB scenario and the (**b**) NB scenario when the output adjustment speed $\omega_{s4}^{\sigma }=0.015,0.027,0.033,0.0343,0.037$.

Figure 8a,b display the attractors of the system’s output decisions under the IB and NB scenarios, respectively, as the output adjustment speed $\omega_{s4}^{\sigma }(\sigma =IB,NB)$ varies. The blue, red, green, magenta, and black dots represent the attractors at output adjustment speeds $\omega_{s4}^{\sigma }$ of 0.015, 0.027, 0.033, 0.0343, and 0.037, respectively. As $\omega_{s4}^{\sigma }$ increases, the attractors shift from a stable point to 2-cycle points, indicating a transition to periodic oscillations and a rise in instability in the system’s output decisions. This suggests that excessive output adjustment speeds destabilize the system. Specifically, for both the IB and NB scenarios, the system loses stability when $\omega_{s4}^{\sigma }$ reaches or exceeds 0.027. Therefore, it is crucial for PM chains under both scenarios to maintain output adjustment speeds below 0.027 to preserve system stability.

**Figure 9:**
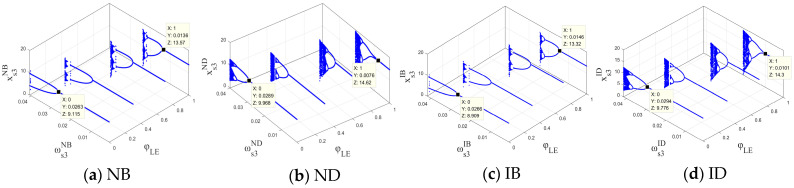
Three-dimensional bifurcation diagrams of the system with respect to $\omega_{s3}^{\sigma }$ and $\varphi_{LE}$ under the (**a**) NB, (**b**) ND, (**c**) IB, and (**d**) ID scenarios.

To explore the impact of the commission rate $\varphi_{LE}$ on the stability of the system’s output decisions, Figure 9 shows the three-dimensional bifurcation diagrams of the PM chain s3’s output decisions as $\varphi_{LE}$, and the output adjustment speed $\omega_{s3}^{\sigma }$ varies under the four scenarios. Under the NB scenario, when the domestic CBEC platform LE’s commission rate $\varphi_{LE}$ is 0%, the bifurcation point at which the system’s output decisions lose stability is $\omega_{s3}^{NB}=$ 0.0263, while at a commission rate of 100%, the bifurcation point is $\omega_{s3}^{NB}=$ 0.0136, as shown in Figure 9a. Thus, under the NB scenario, as the commission rate $\varphi_{LE}$ increases from 0 to 100%, the system’s output decisions regarding $\omega_{s3}^{\sigma }$ enter a bifurcated state and lose stability 0.0127 units earlier. Under the ND, IB, and ID scenarios, these advances are 0.0213, 0.012, and 0.0193, respectively, as shown in Figure 9b–d.

Therefore, under the four scenarios, as the commission rate $\varphi_{LE}$ increases, the bifurcation points for the system’s output decisions regarding $\omega_{s3}^{\sigma }$ occur earlier, and the stable range for $\omega_{s3}^{\sigma }$ is compressed. Furthermore, the comparison of the bifurcation points under the four scenarios—NB, ND, IB, and ID—shows the following values: 0.0127, 0.0213, 0.012, and 0.0193, respectively. This indicates that the direct-mail logistics mode (ND and ID) can improve the stability of the system’s output decisions regarding $\omega_{s3}^{\sigma }$ when the commission rate $\varphi_{LE}$ increases. Additionally, adopting the information-sharing strategy (IB and ID) also helps maintain the stability of the system’s output decisions regarding $\omega_{s3}^{\sigma }$ as the commission rate $\varphi_{LE}$ increases.

### 5.2. The Impact of Tariff on System Stability

**Figure 10:**
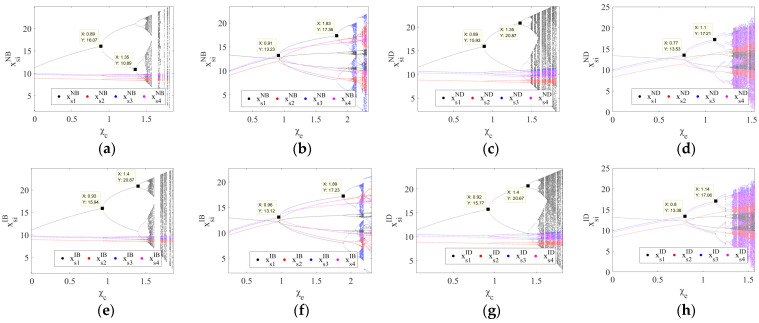
Bifurcation diagrams of the system with respect to $\chi_{c}$ under the (**a**) NB, (**c**) ND, (**e**) IB, and (**g**) ID scenarios. Bifurcation diagrams of the system with respect to $\chi_{e}$ under the (**b**) NB, (**d**) ND, (**f**) IB, and (**h**) ID scenarios.

To investigate the impact of tariffs on system stability, Figure 10 shows the bifurcation diagrams of the system’s output decisions under four scenarios as the general import comprehensive tax rate $\chi_{c}$ and the CBEC comprehensive tax rate $\chi_{e}$ vary. Under the NB scenario, the system loses stability and begins to exhibit bifurcation phenomena when the general import comprehensive tax rate $\chi_{c}$ is 0.89 or when the CBEC comprehensive tax rate $\chi_{e}$ reaches 0.91, as shown in Figure 10a,b. Under the ND scenario, bifurcation occurs when the general import comprehensive tax rate $\chi_{c}$ is 0.89 or when the CBEC comprehensive tax rate $\chi_{e}$ is 0.77, as depicted in Figure 10c,d. Under the IB scenario, the system loses stability and subsequently enters a two-cycle state when the general import comprehensive tax rate $\chi_{c}$ is 0.93 or when the CBEC comprehensive tax rate $\chi_{e}$ reaches 0.96, as shown in Figure 10e,f. Under the ID scenario, bifurcation phenomena occur when the general import comprehensive tax rate $\chi_{c}$ is 0.92 or when the CBEC comprehensive tax rate $\chi_{e}$ is 0.8, as shown in Figure 10g,h. This indicates that the tax rate of the importing country plays a crucial role in the stability of the system’s output decisions. Under the four studied scenarios, the system loses stability when the general import comprehensive tax rate $\chi_{c}$ and the CBEC comprehensive tax rate $\chi_{e}$ each exceed specific thresholds. Therefore, the importing countries should carefully consider the setting of tax rates and ensure that they do not exceed the aforementioned critical thresholds, thus ensuring the stable operation of the supply chain system.

A comparison of the threshold values for tax rates under the four scenarios demonstrates that different logistics modes have different stability. When the overseas manufacturer $a_{2}$ adopts the bonded warehouse mode (NB and IB), the stable range for the system’s output decisions regarding the CBEC comprehensive tax rate $\chi_{e}$ is larger, indicating that the bonded warehouse mode (NB and IB) may have a stronger adaptive capacity to changes in the CBEC comprehensive tax rate $\chi_{e}$. Under the direct-mail mode (ND and ID), the stable range for the system’s output decisions regarding the general import comprehensive tax rate $\chi_{c}$ is larger and, so, the direct-mail mode (ND and ID) is more stable in the face of changes in the general import comprehensive tax rate $\chi_{c}$. This implies that the bonded warehouse mode (NB and IB) may exhibit greater stability against fluctuations in the CBEC comprehensive tax rate $\chi_{e}$ due to its operational characteristics, such as tax incentives and inventory management. In contrast, the direct-mail mode (ND and ID) may demonstrate higher stability with respect to the general import comprehensive tax rate $\chi_{c}$, potentially due to its simplified logistics processes and lower inventory risks. Furthermore, a comparison of the bifurcation diagrams under the ND and ID scenarios, as well as the NB and IB scenarios (as shown in Figure 10), reveals that, regardless of whether the overseas manufacturer $a_{2}$ chooses the direct-mail mode or the bonded warehouse mode, the information-sharing strategy of the domestic CBEC platform LE can significantly enhance the stability of the system’s output decisions with respect to both the general import comprehensive tax rate $\chi_{c}$ and the CBEC comprehensive tax rate $\chi_{e}$.

**Figure 11:**
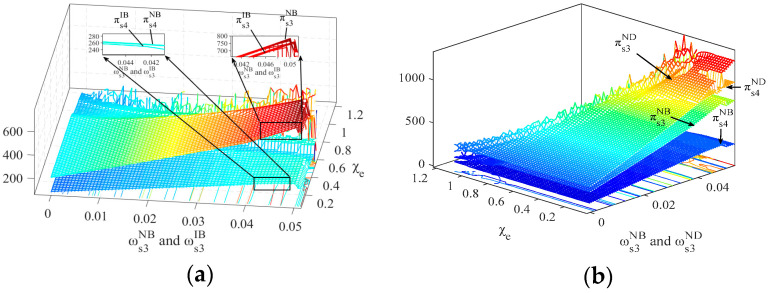
Three-dimensional diagrams of the profits of PM chains s3 and s4 with respect to $\omega_{s3}^{\sigma }$ and $\chi_{e}$ under the (**a**) NB and IB scenarios; (**b**) NB and ND scenarios.

From the analysis above, we can conclude that the output adjustment speed and the tariff rate are two key factors affecting the stability of output decisions of the PM chain. Therefore, their variations are directly related to the profit stability of the PM chain. Figure 11 shows the impacts of increases in the output adjustment speed $\omega_{s3}^{\sigma }$ and the CBEC comprehensive tax rate $\chi_{e}$ on the profits of PM chains s3 and s4. Specifically, Figure 10a compares the profits of s3 and s4 under non-information sharing or information sharing (NB and IB), while Figure 11b contrasts the profits between bonded warehouse or direct-mail logistics modes (NB and ND). From Figure 11a, we can observe that as the CBEC comprehensive tax rate $\chi_{e}$ increases, when the domestic CBEC platform LE adopts a non-information-sharing strategy, the profit levels of PM chains s3 and s4 are higher than with information sharing. Figure 11b shows that when the overseas manufacturer $a_{2}$ chooses the direct-mail mode, PM chains s3 and s4 can achieve higher profits. This phenomenon suggests that the non-information-sharing strategy and the direct-mail logistics mode may be more conducive to enhancing the profit levels of the overseas manufacturer $a_{2}$. Furthermore, as the CBEC comprehensive tax rate $\chi_{e}$ increases, the profits of PM chains s3 and s4 gradually lose their stability and tend to fluctuate. Therefore, a high CBEC comprehensive tax rate set by the importing country not only disrupts the stability of the system’s output decisions but also suppresses the profits of the overseas manufacturer.

### 5.3. Chaos Control of the System

When the CBEC market is in a chaotic state, the output decisions of each PM chain in the system are uncertain, and the PM chains are unable to achieve profit maximization. Therefore, it is necessary to control the chaotic phenomena in the system’s output decisions. This section employs the delay feedback control method to control the bifurcation and chaotic states of the output decisions in the closed-loop supply chain system of CBEC. That is, each PM chain fully considers the difference between its current output decision $x_{si,t}^{\sigma }$ and the expected output decision $x_{si,t+1}^{\sigma }$ when making the next stage’s output decision and adjusts the next stage’s output decision $x_{si,t+1}^{\sigma }$ based on this difference [36]. Let the control factor $\delta^{\sigma }$ represent the PM chain si’s reference level for the difference between the current and expected output decisions. Then, under the delay feedback control method, the adjustment process of the PM chain si’s output decision becomes $x_{si,t+1}^{\sigma }=x_{si,t}^{\sigma }+\omega_{si}^{\sigma }x_{si,t}^{\sigma }\frac{\partial \pi_{si,t}^{\sigma }}{\partial x_{si,t}^{\sigma }}+T_{si}^{\sigma }$, where $T_{si}^{\sigma }=\delta^{\sigma }(x_{si,t}^{\sigma }-x_{si,t+1}^{\sigma })$. Taking the NB scenario as an example, the delay feedback control model of the closed-loop supply chain system of CBEC can be expressed in Equations (39) and (40).

(40) $$\begin{array}{l} x_{s1,t+1}^{NB}=x_{s1,t}^{NB}+\omega_{s1}^{NB}x_{s1,t}^{NB}((E(u_{x})-2x_{s1,t}^{NB}-\beta (x_{s2,t}^{NB}+x_{s3,t}^{NB}+x_{s4,t}^{NB}))(1+\varphi_{CE})(1+\chi_{c})-2\gamma_{1}x_{s1,t}^{NB}+\gamma_{2}q_{s1,t}^{NB}-\gamma_{4})+T_{s1}^{NB}\\ x_{s2,t+1}^{NB}=x_{s2,t}^{NB}+\omega_{s2}^{NB}x_{s2,t}^{NB}((u-2x_{s2,t}^{NB}-\beta (x_{s1,t}^{NB}+x_{s3,t}^{NB}+x_{s4,t}^{NB}))(1+\chi_{e})\Delta_{s2}-2\gamma_{6}x_{s2,t}^{NB}+\gamma_{7}q_{s2,t}^{NB}-\gamma_{9})+T_{s2}^{NB}\\ x_{s3,t+1}^{NB}=x_{s3,t}^{NB}+\omega_{s3}^{NB}x_{s3,t}^{NB}((E(u_{x})-2x_{s3,t}^{NB}-\beta (x_{s1,t}^{NB}+x_{s2,t}^{NB}+x_{s4,t}^{NB}))(1+\varphi_{LE})(1+\chi_{e})\Delta_{s3}-2\gamma_{11}x_{s3,t}^{NB}+\gamma_{12}q_{s3,t}^{NB}-\gamma_{14})+T_{s3}^{NB}\\ x_{s4,t+1}^{NB}=x_{s4,t}^{NB}+\omega_{s4}^{NB}x_{s4,t}^{NB}((E(u_{x})-2x_{s4,t}^{NB}-\beta (x_{s1,t}^{NB}+x_{s2,t}^{NB}+x_{s3,t}^{NB}))(1+\varphi_{LE})(1+\chi_{e})\Delta_{s4}-2\gamma_{16}x_{s4,t}^{NB}+\gamma_{17}q_{s4,t}^{NB}-\gamma_{19})+T_{s4}^{NB}\\ q_{s1,t+1}^{NB}=x_{s1,t}^{NB}\\ q_{s2,t+1}^{NB}=x_{s2,t}^{NB}\\ q_{s3,t+1}^{NB}=x_{s3,t}^{NB}\\ q_{s4,t+1}^{NB}=x_{s4,t}^{NB} \end{array}$$

**Figure 12:**
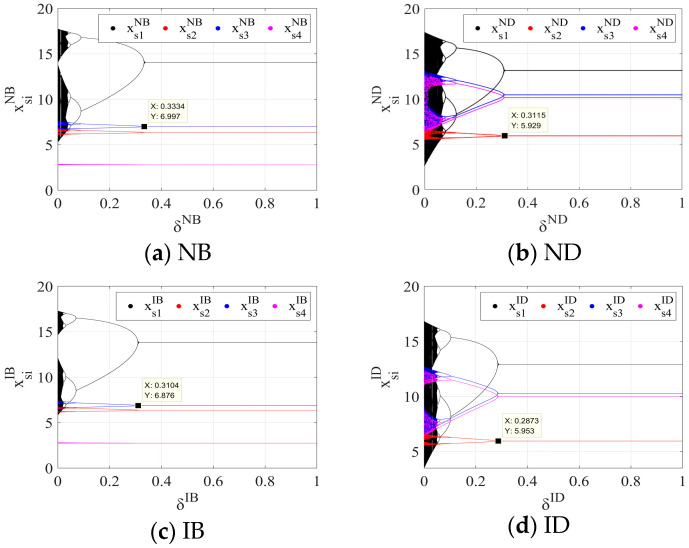
Bifurcation diagrams of the system after chaos control under the (**a**) NB, (**b**) ND, (**c**) IB, and (**d**) ID scenarios.

Figure 12 shows the impact of the control factor $\delta^{\sigma }$ on the system’s output decision-making under four scenarios. As the control factor $\delta^{\sigma }$ increases, the system’s output decisions gradually transition from a chaotic state back to a stable state. This indicates that under four scenarios, the PM chain can effectively control the chaotic state of output decisions by increasing the reference level to the difference between current $x_{si,t}^{\sigma }$ and expected output decisions $x_{si,t+1}^{\sigma }$. Under the NB, ND, IB, and ID scenarios, the unstable states of the system’s output decisions are effectively suppressed at $\delta^{NB}=0.3334$, $\delta^{ND}=0.3115$, $\delta^{IB}=0.3104$, and $\delta^{ID}=0.2873$, respectively, and the system’s output decisions return to stability. Therefore, under the four scenarios, the PM chain should ensure that the reference level to the difference between current $x_{si,t}^{\sigma }$ and expected output decisions $x_{si,t+1}^{\sigma }$ exceeds the aforementioned thresholds. Moreover, the comparison of the thresholds across the four scenarios shows that the NB scenario requires the largest control factor $\delta^{NB}$ to achieve the transition from a chaotic state to a stable state, while the ID scenario requires the smallest control factor $\delta^{ID}$. This suggests that the information-sharing strategy and direct-mail mode can significantly enhance the system’s ability to restore stability. Therefore, to effectively respond to market fluctuations and maintain the stability of output decisions, the domestic CBEC platform LE should strengthen its information-sharing mechanisms. Meanwhile, the overseas manufacturer $a_{2}$ should flexibly choose the direct-mail logistics mode based on an in-depth analysis of product characteristics and market demands.

**Figure 13:**
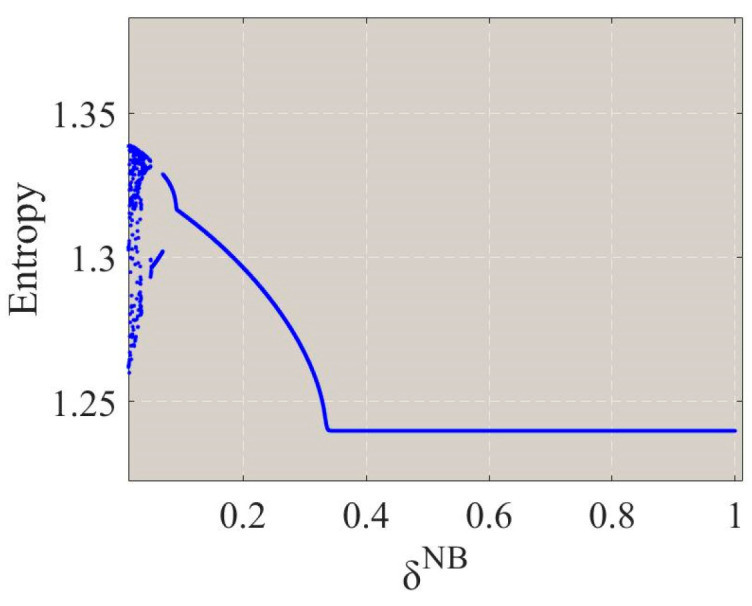
Entropy diagram of the system under the NB scenario.

Similarly, Figure 13 shows that as the control factor $\delta^{NB}$ increases, the entropy value of the system’s output decisions decreases, and the stability of the system’s output decision increases.

### 5.4. Sensitivity Analysis of System Stability

In summary, maintaining the stability of the system’s output decisions requires controlling the output adjustment speed and tariff rates of the PM chain within specific thresholds. To further explore the variations in these thresholds under changes in other parameter values, a sensitivity analysis was conducted regarding the system’s output decisions with respect to the output adjustment speed $\omega_{s3}^{NB}$, the general import comprehensive tax rate $\chi_{c}$, and the CBEC comprehensive tax rate $\chi_{e}$, taking the NB scenario as an example. Under real market environments, differences in the product price u and channel substitutability rate β significantly influence consumer demand, thereby altering the output decisions of supply chain members and impacting the long-term dynamic evolution of the supply chain. Therefore, this study performed a sensitivity analysis on u and β in order to investigate their effects on the dynamic changes in system output decisions, as detailed in Table 2.

(1) Sensitivity analysis on u: As u increases from 20 to 65, the period-doubling bifurcation points for $\omega_{s3}^{NB}$, $\chi_{c}$, and $\chi_{e}$ are delayed from 0.0233, 0.82, and 0.79 to 0.0242, 0.88, and 0.91, respectively. This indicates that, for higher-priced products, the closed-loop supply chain system of CBEC exhibits a broader stability range in terms of output decisions and tax rates. This finding suggests that higher-end products—which are typically associated with higher prices and profit margins—enable supply chain members to maintain more stable output decisions, thereby reducing sensitivity to fluctuations in consumer demand. In terms of tariff policies, the high value of premium products allows them to withstand greater cost impacts from changes in CBEC and regular import tariffs. Even with tax rate adjustments, the overall cost structure is relatively less affected, reducing the sensitivity of supply chain members to tax policy changes. This stability not only helps supply chain members develop more long-term strategic plans but also promotes resilience against uncertainties in the external policy environment.

(2) Sensitivity analysis on β: As β increases from 0.1 to 0.7, the period-doubling bifurcation points for $\omega_{s3}^{NB}$, $\chi_{c}$, and $\chi_{e}$ are delayed from 0.0213, 0.69, and 0.59 to 0.0273, 1.07, and 1.16, respectively. This indicates that higher channel substitutability enhances system stability. Specifically, an increase in channel substitutability implies that different sales channels become more interchangeable, providing consumers with greater flexibility and diversity in their purchasing decisions. In terms of output decisions, higher channel substitutability allows supply chain members to respond more effectively to market demand fluctuations when carrying out production planning. Even if demand decreases in one channel, other channels can compensate for the shortfall, thereby reducing the risk of production shocks caused by fluctuations in a single channel’s demand. For tariff policies, higher channel substitutability also enhances the adaptability of supply chain members to tariff rate changes: if the tariff rate for one channel increases, consumers are likely to switch to other channels, mitigating the impact of tariff adjustments on the cost structure of supply chain members. This adaptability allows supply chain members to formulate tariff strategies with greater flexibility and resilience.

To demonstrate the universality of the proposed model, we selected two representative products with different prices—tablet computers (whose prices are higher than those of wireless telephones) and mobile phone chargers (whose prices are lower than those of wireless telephones)—for numerical simulation and analysis. The analysis results indicated that the dynamic evolution of the system’s output decisions for both products was similar to the example of wireless telephones.

## 6. Conclusions

This study, from the perspective of inter-chain competition, is the first to investigate the multi-stage dynamic evolution and stability of output decisions in the closed-loop supply chain system of CBEC. By constructing discrete dynamic models for four product–market (PM) chains, we not only revealed the dynamic evolution patterns of production decisions under different scenarios but also analyzed the impacts of tax policies on system stability. The main findings and contributions of this study are summarized as follows:

(1) **Threshold analysis for output decision stability**: We found that the closed-loop supply chain system of CBEC can achieve stability only when the initial output decisions and adjustment speeds remain within specific threshold ranges. Otherwise, the system transitions to chaos or uncontrollability through period-doubling bifurcations. This discovery offers a new perspective for supply chain management, emphasizing that overseas manufacturers must account for the long-term impacts of initial production decisions and adjustment speeds on system stability, ensuring that these factors remain below the critical thresholds.

(2) **Impact of tariff policies on system stability:** This study is the first to quantify the specific effects of tariff rate changes on the stability of the closed-loop supply chain system of CBEC. It identified the thresholds for the general trade and CBEC import comprehensive tax rates under which the system remains stable. When these rates exceed their respective thresholds, the system’s output decisions and PM chain profits become unstable. These findings provide critical guidance for policymakers, suggesting that import governments should carefully consider these thresholds when formulating tariff policies.

(3) **Comparison of logistics mode choices by overseas suppliers**: For the first time, we compared bonded warehouse and direct-mail logistics modes under different tax rates. We found that the direct-mail mode is more stable in the face of changes in the general trade import comprehensive tax rate, while the bonded warehouse mode is more adaptable to fluctuations in the CBEC import comprehensive tax rate. This insight provides overseas manufacturers with new decision-making criteria when selecting logistics modes in response to tariff changes and encourages policymakers to account for the differing adaptabilities of logistics modes to tariff rate changes.

(4) **Effects of information-sharing strategies and commission rates on system stability**: This study revealed the significant impacts of information-sharing strategies on supply chain stability within CBEC platforms. Information sharing can significantly expand the region of stability for output decisions, thereby enhancing overall system stability. This finding offers a novel strategic tool for CBEC platforms to improve supply chain stability. Additionally, the study revealed that as commission rates increase, the bifurcation points of output adjustment speeds appear earlier, reducing the stable region for output decisions and thus diminishing system stability. This suggests that setting lower commission rates may help to maintain system stability, providing a new perspective on how platforms can influence supply chain stability through commission adjustments.

(5) **Application of chaos control methods**: For the first time, we successfully applied delayed feedback control to manage the closed-loop supply chain system of CBEC. This method effectively controls chaotic states, restoring output decision stability from fluctuating conditions. When the market enters a chaotic state, overseas suppliers can use delayed feedback control to manage their output decisions and restore stability. This application not only offers a new solution for supply chain management but also provides a practical example of chaos control theory in real-world business environments.

(6) **Sensitivity of system stability thresholds to output decisions and tax rates**: The stability thresholds of the considered system are highly sensitive to product market prices and the substitutability of PM chain channels. When products exhibit higher market prices or stronger channel substitutability, the output decisions of the closed-loop supply chain system of CBEC demonstrate larger stability thresholds and stronger stability with respect to output adjustment speeds and tax rates.

As the first study to explore the stability of decision-making in a closed-loop supply chain system of CBEC from the perspective of inter-chain competition, we focused on the unidirectional export scenario; that is, from overseas manufacturers to the domestic market. Future research could be extended to more complex scenarios, such as exploring bidirectional trade interactions between CBEC supply chains in two countries.

## Figures and Tables

**Table 1:** Key notations.

Notations	Descriptions
**Symbols:**	
si	The PM chains, i=1,2,3,4 The links in PM chains, m=1,2,…,17
$b_{m}$	
t	Period t
σ∈{NB,ND,IB,ID}	Superscript, index of the four scenarios (non-information-sharing and bonded warehouse mode, non-information-sharing strategy and direct-mail mode, information-sharing and bonded warehouse mode, information-sharing and direct-mail mode)
$\varepsilon \in \left\{a_{1},a_{2},CE,LE,IL,TL,PL,BW,RB\right\}$	Subscript, index of the overseas raw material supplier, overseas manufacturer, overseas CBEC platform, domestic CBEC platform, overseas international logistic company, the domestic third-party logistic company, the domestic platforms’ self-built logistics company, bonded warehouses, and recycler
**Parameters:**	
β	The substitutability rate of product $P^{*}$ sold by overseas manufacturer $a_{2}$ through different channels
u	The market price of product $P^{*}$ in the market MR
$E(u_{x})$	The expected market price of product $P^{*}$
$\chi_{c}$	The general trade import comprehensive tax rate
$\chi_{e}$	The CBEC import comprehensive tax rate
$\varphi_{LE}$	The commission rate of domestic CBEC platform LE for product $P^{*}$
$\varphi_{CE}$	The commission rate of overseas CBEC platform CE for product $P^{*}$
$\rho_{\varepsilon }$	The quantity of materials required by companies $a_{1}$ and $a_{2}$ to manufacture one unit of final product $P^{*}$, $\varepsilon \in \left\{a_{1},a_{2}\right\}$
$\Delta_{si}$	The actual transaction ratio of product $P^{*}$ in each period of the PM chain si
υ	The recycling rate of $P^{*}$ in the market
η	The proportion of recycled products that can be re-manufactured
$r_{\varepsilon }$	The marginal production, operation, storage, or re-manufacturing cost of product $P^{*}$ for company ε
$r_{a_{1}}^{-}$	The marginal production cost of re-manufactured products for overseas raw material supplier $a_{1}$
$h_{RB}$	The marginal disposal cost of waste products by recycler RB
$\omega_{si}^{NB}$	The output adjustment speed of PM chain si
$\delta^{\sigma }$	The control factors for output decisions under scenario σ
**Functions:**	
$P_{si,t}^{\sigma }$	The price of product $P^{*}$ for PM chain si under scenario σ
$f_{\varepsilon si,t}^{\sigma }$	The production or operational quantity of product $P^{*}$ by company ε in the PM chain si
$l_{b_{m}si,t}^{\sigma }$	The material flow of product $P^{*}$ on link $b_{m}$ in the PM chain si
$C_{\varepsilon si,t}^{\sigma }$	The total production or operating cost of company ε in PM chain si for product $P^{*}$
$C_{b_{m}si,t}^{\sigma }$	The transaction cost of link $b_{m}$ in PM chain si for product $P^{*}$
$C_{si,t}^{\sigma }$	The total cost of PM chain si under scenario σ
$\pi_{si,t}^{\sigma }$	The profit of PM chain si under scenario σ
**Decision variables:**	
$x_{si,t}^{\sigma }$	In period t, the output decisions of PM chain si for product $P^{*}$ under scenario σ

**Table 2:** Sensitivity analysis of the bifurcation points of the system’s output decisions under the NB Scenario.

Parameters	Values	Bifurcation Points of the Two-Cycle State with Respect to $\omega_{s3}^{NB}$	Bifurcation Points of the Two-Cycle State with Respect to $\chi_{c}$	Bifurcation Points of the Two-Cycle State with Respect to $\chi_{e}$
u	65	0.0242	0.88	0.91
	50	0.0239	0.87	0.90
	35	0.0236	0.85	0.84
	20	0.0233	0.82	0.79
β	0.7	0.0273	1.07	1.16
	0.5	0.0253	0.96	1.01
	0.3	0.0233	0.83	0.83
	0.1	0.0213	0.69	0.59

## Data Availability

Data are contained within the article.

## Appendix A

The concrete expressions of $\gamma_{1}-\gamma_{27}$:

(A1) $$\gamma_{1}=r_{a_{1}}{\rho_{a_{1}}}^{2}+r_{a_{2}}{\rho_{a_{2}}}^{2}+r_{b_{1}}{\rho_{a_{1}}}^{2}+r_{b_{2}}{\rho_{a_{2}}}^{2}+r_{b_{3}}+r_{b_{4}},\gamma_{2}=2r_{a_{1}}{\rho_{a_{1}}}^{2}\eta \upsilon$$

(A2) $$\gamma_{3}=r_{a_{1}}{\rho_{a_{1}}}^{2}\eta^{2}\upsilon^{2}+r_{a_{1}}^{-}{\rho_{a_{1}}}^{2}\eta^{2}\upsilon^{2}+r_{RB}\eta^{2}\upsilon^{2}+r_{b_{15}}\upsilon^{2}+r_{b_{16}}\upsilon^{2}+r_{b_{17}}\upsilon^{2}+r_{b_{18}}\eta^{2}\upsilon^{2},\gamma_{4}=r_{CE}+r_{IL}$$

(A3) $$\gamma_{5}=r_{PL}\upsilon +r_{LE}\upsilon +h_{RB}(1-\eta )\upsilon ,\gamma_{6}=r_{a_{1}}{\rho_{a_{1}}}^{2}+r_{a_{2}}{\rho_{a_{2}}}^{2}+r_{b_{1}}{\rho_{a_{1}}}^{2}+r_{b_{5}}{\rho_{a_{2}}}^{2}+r_{b_{6}}{\rho_{a_{2}}}^{2}+r_{b_{7}}{\Delta_{s2}}^{2}+r_{b_{8}}{\Delta_{s2}}^{2}$$

(A4) $$\gamma_{7}=2(r_{a_{1}}{\rho_{a_{1}}}^{2}(1-\Delta_{s2}+\eta \upsilon \Delta_{s2})+r_{a_{2}}{\rho_{a_{2}}}^{2}(1-\Delta_{s2})+r_{b_{1}}{\rho_{a_{1}}}^{2}(1-\Delta_{s2})+r_{b_{5}}{\rho_{a_{2}}}^{2}(1-\Delta_{s2})+r_{b_{6}}{\rho_{a_{2}}}^{2}(1-\Delta_{s2}))$$

(A5) $$\begin{array}{ll} \gamma_{8} & =r_{a_{1}}{\rho_{a_{1}}}^{2}{\left(1-\Delta_{s2}+\eta \upsilon \Delta_{s2}\right)}^{2}+r_{a_{1}}^{-}{\rho_{a_{1}}}^{2}\eta^{2}\upsilon^{2}{\Delta_{s2}}^{2}+r_{a_{2}}{\rho_{a_{2}}}^{2}{\left(1-\Delta_{s2}\right)}^{2}+r_{RB}\eta^{2}\upsilon^{2}{\Delta_{s2}}^{2}+r_{b_{1}}{\rho_{a_{1}}}^{2}{\left(1-\Delta_{s2}\right)}^{2}+\\ & r_{b_{5}}{\rho_{a_{2}}}^{2}{\left(1-\Delta_{s2}\right)}^{2}+r_{b_{6}}{\rho_{a_{2}}}^{2}{\left(1-\Delta_{s2}\right)}^{2}+(r_{b_{15}}+r_{b_{16}}+r_{b_{17}}+r_{b_{18}}\eta^{2})\upsilon^{2}{\Delta_{s2}}^{2} \end{array}$$

(A6) $$\gamma_{9}=r_{LE}+r_{PL}\Delta_{s2}+r_{BW},\gamma_{10}=(r_{LE}+r_{PL}+h_{RB}(1-\eta ))\upsilon \Delta_{s2}$$

(A7) $$\gamma_{11}=r_{a_{1}}{\rho_{a_{1}}}^{2}+r_{a_{2}}{\rho_{a_{2}}}^{2}+r_{b_{1}}{\rho_{a_{1}}}^{2}+r_{b_{9}}{\rho_{a_{2}}}^{2}+r_{b_{10}}{\rho_{a_{2}}}^{2}+r_{b_{11}}{\Delta_{s3}}^{2}+r_{b_{12}}{\Delta_{s3}}^{2}$$

(A8) $$\gamma_{12}=2(r_{a_{1}}{\rho_{a_{1}}}^{2}(1-\Delta_{s3}+\eta \upsilon \Delta_{s3})+r_{a_{2}}{\rho_{a_{2}}}^{2}(1-\Delta_{s3})+r_{b_{1}}{\rho_{a_{1}}}^{2}(1-\Delta_{s3})+r_{b_{9}}{\rho_{a_{2}}}^{2}(1-\Delta_{s3})+r_{b_{10}}{\rho_{a_{2}}}^{2}(1-\Delta_{s3}))$$

(A9) $$\begin{array}{ll} \gamma_{13} & =r_{a_{1}}{\rho_{a_{1}}}^{2}{\left(1-\Delta_{s3}+\eta \upsilon \Delta_{s3}\right)}^{2}+r_{a_{1}}^{-}{\rho_{a_{1}}}^{2}\eta^{2}\upsilon^{2}{\Delta_{s3}}^{2}+r_{a_{2}}{\rho_{a_{2}}}^{2}{\left(1-\Delta_{s3}\right)}^{2}+r_{RB}\eta^{2}\upsilon^{2}{\Delta_{s3}}^{2}+r_{b_{1}}{\rho_{a_{1}}}^{2}{\left(1-\Delta_{s3}\right)}^{2}+\\ & r_{b_{9}}{\rho_{a_{2}}}^{2}{\left(1-\Delta_{s3}\right)}^{2}+r_{b_{10}}{\rho_{a_{2}}}^{2}{\left(1-\Delta_{s3}\right)}^{2}+(r_{b_{15}}+r_{b_{16}}+r_{b_{17}}+r_{b_{18}}\eta^{2})\upsilon^{2}{\Delta_{s3}}^{2} \end{array}$$

(A10) $$\gamma_{14}=r_{LE}+r_{PL}\Delta_{s3}+r_{BW},\gamma_{15}=r_{LE}\upsilon \Delta_{s3}+r_{PL}\upsilon \Delta_{s3}+h_{RB}(1-\eta )\upsilon \Delta_{s3}$$

(A11) $$\gamma_{16}=r_{a_{1}}{\rho_{a_{1}}}^{2}+r_{a_{2}}{\rho_{a_{2}}}^{2}+r_{b_{1}}{\rho_{a_{1}}}^{2}+r_{b_{9}}{\rho_{a_{2}}}^{2}+r_{b_{10}}{\rho_{a_{2}}}^{2}+r_{b_{13}}{\Delta_{s4}}^{2}+r_{b_{14}}{\Delta_{s4}}^{2}$$

(A12) $$\gamma_{17}=2(r_{a_{1}}{\rho_{a_{1}}}^{2}(1-\Delta_{s4}+\eta \upsilon \Delta_{s4})+r_{a_{2}}{\rho_{a_{2}}}^{2}(1-\Delta_{s4})+r_{b_{1}}{\rho_{a_{1}}}^{2}(1-\Delta_{s4})+r_{b_{9}}{\rho_{a_{2}}}^{2}(1-\Delta_{s4})+r_{b_{10}}{\rho_{a_{2}}}^{2}(1-\Delta_{s4}))$$

(A13) $$\begin{array}{ll} \gamma_{18} & =r_{a_{1}}{\rho_{a_{1}}}^{2}{\left(1-\Delta_{s4}+\eta \upsilon \Delta_{s4}\right)}^{2}+r_{a_{1}}^{-}{\rho_{a_{1}}}^{2}\eta^{2}\upsilon^{2}{\Delta_{s4}}^{2}+r_{a_{2}}{\rho_{a_{2}}}^{2}{\left(1-\Delta_{s4}\right)}^{2}+r_{RB}\eta^{2}\upsilon^{2}{\Delta_{s4}}^{2}+r_{b_{1}}{\rho_{a_{1}}}^{2}{\left(1-\Delta_{s4}\right)}^{2}+\\ & r_{b_{9}}{\rho_{a_{2}}}^{2}{\left(1-\Delta_{s4}\right)}^{2}+r_{b_{10}}{\rho_{a_{2}}}^{2}{\left(1-\Delta_{s4}\right)}^{2}+r_{b_{15}}\upsilon^{2}{\Delta_{s4}}^{2}+r_{b_{16}}\upsilon^{2}{\Delta_{s4}}^{2}+r_{b_{17}}\upsilon^{2}{\Delta_{s4}}^{2}+r_{b_{18}}\eta^{2}\upsilon^{2}{\Delta_{s4}}^{2} \end{array}$$

(A14) $$\begin{array}{l} \gamma_{19}=r_{LE}+r_{TL}\Delta_{s4}+r_{BW},\gamma_{20}=r_{LE}\upsilon \Delta_{s4}+r_{PL}\upsilon \Delta_{s4}+h_{RB}(1-\eta )\upsilon \Delta_{s4},\\ \gamma_{21}=r_{a_{1}}{\rho_{a_{1}}}^{2}+r_{a_{2}}{\rho_{a_{2}}}^{2}+r_{b_{1}}{\rho_{a_{1}}}^{2}+r_{b_{9}}{\rho_{a_{2}}}^{2}+r_{b_{10}}+r_{b_{11}} \end{array}$$

(A15) $$\begin{array}{l} \gamma_{22}=2r_{a_{1}}{\rho_{a_{1}}}^{2}\eta \upsilon ,\gamma_{23}=r_{a_{1}}{\rho_{a_{1}}}^{2}\eta^{2}\upsilon^{2}+r_{a_{1}}^{-}{\rho_{a_{1}}}^{2}\eta^{2}\upsilon^{2}+r_{RB}\eta^{2}\upsilon^{2}+\\ r_{b_{14}}\upsilon^{2}+r_{b_{15}}\upsilon^{2}+r_{b_{16}}\upsilon^{2}+r_{b_{17}}\eta^{2}\upsilon^{2},\gamma_{24}=r_{LE}+r_{PL} \end{array}$$

(A16) $$\gamma_{25}=r_{LE}\upsilon +r_{PL}\upsilon +h_{RB}(1-\eta )\upsilon ,\gamma_{26}=r_{a_{1}}{\rho_{a_{1}}}^{2}+r_{a_{2}}{\rho_{a_{2}}}^{2}+r_{b_{1}}{\rho_{a_{1}}}^{2}+r_{b_{9}}{\rho_{a_{2}}}^{2}+r_{b_{12}}+r_{b_{13}},\gamma_{27}=r_{LE}+r_{TL}$$

## Appendix B

The material flow in links $b_{m},m=1,5,6,7,8,15,16,17,18$ for PM chain s2 is given by the following:

(A17) $$\begin{array}{l} l_{b_{1}s2,t}^{NB}=f_{a_{1}s2,t}^{NB};l_{b_{5}s2,t}^{NB}=f_{a_{2}s2,t}^{NB};l_{b_{6}s2,t}^{NB}=x_{si,t}^{NB};l_{b_{7}s2,t}^{NB}=l_{b_{8}s2,t}^{NB}=\Delta_{s2}x_{s2,t}^{NB};\\ l_{b_{15}s2,t}^{NB}=l_{b_{16}s2,t}^{NB}=l_{b_{17}s2,t}^{NB}=\upsilon \Delta_{s2}x_{s2,t-1}^{NB};l_{b_{18}s2,t}^{NB}=f_{RBs2,t}^{NB} \end{array}$$

The material flow in links $b_{m},m=1,9,10,13,14,15,16,17,18$ for PM chains s3 is given by the following:

(A18) $$\begin{array}{l} l_{b_{1}s3,t}^{NB}=f_{a_{1}s3,t}^{NB};l_{b_{9}s3,t}^{NB}=f_{a_{2}s3,t}^{NB};l_{b_{10}s3,t}^{NB}=x_{s3,t}^{NB};l_{b_{11}s3,t}^{NB}=l_{b_{12}s3,t}^{NB}=\Delta_{s3}x_{s3,t}^{NB};\\ l_{b_{15}s3,t}^{NB}=l_{b_{16}s3,t}^{NB}=l_{b_{17}s3,t}^{NB}=\upsilon \Delta_{s3}x_{s3,t-1}^{NB};l_{b_{18}s3,t}^{NB}=f_{RBs3,t}^{NB} \end{array}$$

The material flow in links $b_{m},m=1,9,10,13,14,15,16,17,18$ for PM chain s4 is given by the following:

(A19) $$\begin{array}{l} l_{b_{1}s4,t}^{NB}=f_{a_{1}s4,t}^{NB};l_{b_{9}s4,t}^{NB}=f_{a_{2}s4,t}^{NB};l_{b_{10}s4,t}^{NB}=x_{s4,t}^{NB};l_{b_{13}s4,t}^{NB}=l_{b_{14}s4,t}^{NB}=\Delta_{s4}x_{s4,t}^{NB};\\ l_{b_{15}s4,t}^{NB}=l_{b_{16}s4,t}^{NB}=l_{b_{17}s4,t}^{NB}=\upsilon \Delta_{s4}x_{s4,t-1}^{NB};l_{b_{18}s4,t}^{NB}=f_{RBs4,t}^{NB} \end{array}$$

## Appendix C

The profit of the four PM chains under the NB scenario can be expressed as follows:

(A20) $$\begin{array}{ll} \pi_{s1,t}^{NB} & =(E(u_{x})-x_{s1,t}^{NB}-\beta (x_{s2,t}^{NB}+x_{s3,t}^{NB}+x_{s4,t}^{NB}))(1+\varphi_{CE})(1+\chi_{c})x_{s1,t}^{NB}-\gamma_{1}{x_{s1,t}^{NB}}^{2}+\gamma_{2}x_{s1,t-1}^{NB}x_{s1,t}^{NB}-\\ & \gamma_{3}{x_{s1,t-1}^{NB}}^{2}-\gamma_{4}x_{s1,t}^{NB}-\gamma_{5}x_{s1,t-1}^{NB}\\ \pi_{s2,t}^{NB} & =(u-x_{s2,t}^{NB}-\beta (x_{s1,t}^{NB}+x_{s3,t}^{NB}+x_{s4,t}^{NB}))(1+\chi_{e})\Delta_{s2}x_{s2,t}^{NB}-\gamma_{6}{x_{s2,t}^{NB}}^{2}+\gamma_{7}x_{s2,t-1}^{NB}x_{s2,t}^{NB}-\gamma_{8}{x_{s2,t-1}^{NB}}^{2}-\\ & \gamma_{9}x_{s2,t}^{NB}-\gamma_{10}x_{s2,t-1}^{NB}\\ \pi_{s3,t}^{NB} & =(E(u_{x})-x_{s3,t}^{NB}-\beta (x_{s1,t}^{NB}+x_{s2,t}^{NB}+x_{s4,t}^{NB}))(1+\varphi_{LE})(1+\chi_{e})\Delta_{s3}x_{s3,t}^{NB}-\gamma_{11}{x_{s3,t}^{NB}}^{2}+\gamma_{12}x_{s3,t-1}^{NB}x_{s3,t}^{NB}-\\ & \gamma_{13}{x_{s3,t-1}^{NB}}^{2}-\gamma_{14}x_{s3,t}^{NB}-\gamma_{15}x_{s3,t-1}^{NB}\\ \pi_{s4,t}^{NB} & =(E(u_{x})-x_{s4,t}^{NB}-\beta (x_{s1,t}^{NB}+x_{s2,t}^{NB}+x_{s3,t}^{NB}))(1+\varphi_{LE})(1+\chi_{e})\Delta_{s4}x_{s4,t}^{NB}-\gamma_{16}{x_{s4,t}^{NB}}^{2}+\gamma_{17}x_{s4,t-1}^{NB}x_{s4,t}^{NB}-\\ & \gamma_{18}{x_{s4,t-1}^{NB}}^{2}-\gamma_{19}x_{s4,t}^{NB}-\gamma_{20}x_{s4,t-1}^{NB} \end{array}$$

The profits for PM chains s3 and s4 under the ND scenario are as follows:

(A21) $$\begin{array}{ll} \pi_{s3,t}^{ND} & =(E(u_{x})-x_{s3,t}^{ND}-\beta (x_{s1,t}^{ND}+x_{s2,t}^{ND}+x_{s4,t}^{ND}))(1+\varphi_{LE})(1+\chi_{e})x_{s3,t}^{ND}-\\ & \gamma_{21}{x_{s3,t}^{ND}}^{2}+\gamma_{22}x_{s3,t-1}^{ND}x_{s3,t}^{ND}-\gamma_{23}{x_{s3,t-1}^{ND}}^{2}-\gamma_{24}x_{s3,t}^{ND}-\gamma_{25}x_{s3,t-1}^{ND}\\ \pi_{s4,t}^{ND} & =(E(u_{x})-x_{s4,t}^{ND}-\beta (x_{s1,t}^{ND}+x_{s2,t}^{ND}+x_{s3,t}^{ND}))(1+\varphi_{LE})(1+\chi_{e})x_{s4,t}^{ND}-\\ & \gamma_{26}{x_{s4,t}^{ND}}^{2}+\gamma_{22}x_{s4,t-1}^{ND}x_{s4,t}^{ND}-\gamma_{23}{x_{s4,t-1}^{ND}}^{2}-\gamma_{27}x_{s4,t}^{ND}-\gamma_{25}x_{s4,t-1}^{ND} \end{array}$$

Under the IB scenario, the profits of the four PM chains are as follows:

(A22) $$\begin{array}{ll} \pi_{s1,t}^{IB} & =(u-x_{s1,t}^{IB}-\beta (x_{s2,t}^{IB}+x_{s3,t}^{IB}+x_{s4,t}^{IB}))(1+\varphi_{CE})(1+\chi_{c})x_{s1,t}^{IB}-\gamma_{1}{x_{s1,t}^{IB}}^{2}+\\ & \gamma_{2}x_{s1,t-1}^{IB}x_{s1,t}^{IB}-\gamma_{3}{x_{s1,t-1}^{IB}}^{2}-\gamma_{4}x_{s1,t}^{IB}-\gamma_{5}x_{s1,t-1}^{IB}\\ \pi_{s2,t}^{IB} & =(u-x_{s2,t}^{IB}-\beta (x_{s1,t}^{IB}+x_{s3,t}^{IB}+x_{s4,t}^{IB}))(1+\chi_{e})\Delta_{s2}x_{s2,t}^{IB}-\gamma_{6}{x_{s2,t}^{IB}}^{2}+\\ & \gamma_{7}x_{s2,t-1}^{IB}x_{s2,t}^{IB}-\gamma_{8}{x_{s2,t-1}^{IB}}^{2}-\gamma_{9}x_{s2,t}^{IB}-\gamma_{10}x_{s2,t-1}^{IB}\\ \pi_{s3,t}^{IB} & =(u-x_{s3,t}^{IB}-\beta (x_{s1,t}^{IB}+x_{s2,t}^{IB}+x_{s4,t}^{IB}))(1+\varphi_{LE})(1+\chi_{e})\Delta_{s3}x_{s3,t}^{IB}-\gamma_{11}{x_{s3,t}^{IB}}^{2}+\\ & \gamma_{12}x_{s3,t-1}^{IB}x_{s3,t}^{IB}-\gamma_{13}{x_{s3,t-1}^{IB}}^{2}-\gamma_{14}x_{s3,t}^{IB}-\gamma_{15}x_{s3,t-1}^{IB}\\ \pi_{s4,t}^{IB} & =(u-x_{s4,t}^{IB}-\beta (x_{s1,t}^{IB}+x_{s2,t}^{IB}+x_{s3,t}^{IB}))(1+\varphi_{LE})(1+\chi_{e})\Delta_{s4}x_{s4,t}^{IB}-\gamma_{16}{x_{s4,t}^{IB}}^{2}+\\ & \gamma_{17}x_{s4,t-1}^{IB}x_{s4,t}^{IB}-\gamma_{18}{x_{s4,t-1}^{IB}}^{2}-\gamma_{19}x_{s4,t}^{IB}-\gamma_{20}x_{s4,t-1}^{IB} \end{array}$$

Under the ID scenario, the profits of the four PM chains are as follows:

(A23) $$\begin{array}{ll} \pi_{s1,t}^{ID} & =(u-x_{s1,t}^{ID}-\beta (x_{s2,t}^{ID}+x_{s3,t}^{ID}+x_{s4,t}^{ID}))(1+\varphi_{CE})(1+\chi_{c})x_{s1,t}^{ID}-\gamma_{1}{x_{s1,t}^{ID}}^{2}+\gamma_{2}x_{s1,t-1}^{ID}x_{s1,t}^{ID}-\\ & \gamma_{3}{x_{s1,t-1}^{ID}}^{2}-\gamma_{4}x_{s1,t}^{ID}-\gamma_{5}x_{s1,t-1}^{ID}\\ \pi_{s2,t}^{ID} & =(u-x_{s2,t}^{ID}-\beta (x_{s1,t}^{ID}+x_{s3,t}^{ID}+x_{s4,t}^{ID}))(1+\chi_{e})\Delta_{s2}x_{s2,t}^{ID}-\gamma_{6}{x_{s2,t}^{ID}}^{2}+\\ & \gamma_{7}x_{s2,t-1}^{ID}x_{s2,t}^{ID}-\gamma_{8}{x_{s2,t-1}^{ID}}^{2}-\gamma_{9}x_{s2,t}^{ID}-\gamma_{10}x_{s2,t-1}^{ID}\\ \pi_{s3,t}^{ID} & =(u-x_{s3,t}^{ID}-\beta (x_{s1,t}^{ID}+x_{s2,t}^{ID}+x_{s4,t}^{ID}))(1+\varphi_{LE})(1+\chi_{e})x_{s3,t}^{ID}-\gamma_{21}{x_{s3,t}^{ID}}^{2}+\\ & \gamma_{22}x_{s3,t-1}^{ID}x_{s3,t}^{ID}-\gamma_{23}{x_{s3,t-1}^{ID}}^{2}-\gamma_{24}x_{s3,t}^{ID}-\gamma_{25}x_{s3,t-1}^{ID}\\ \pi_{s4,t}^{ID} & =(u-x_{s4,t}^{ID}-\beta (x_{s1,t}^{ID}+x_{s2,t}^{ID}+x_{s3,t}^{ID}))(1+\varphi_{LE})(1+\chi_{e})x_{s4,t}^{ID}-\gamma_{26}{x_{s4,t}^{ID}}^{2}+\\ & \gamma_{22}x_{s4,t-1}^{ID}x_{s4,t}^{ID}-\gamma_{23}{x_{s4,t-1}^{ID}}^{2}-\gamma_{27}x_{s4,t}^{ID}-\gamma_{25}x_{s4,t-1}^{ID} \end{array}$$

## Appendix D

The concrete expression of $B_{0}^{\sigma }$–$B_{7}^{\sigma }$ in the characteristic polynomial (21):

(A24) $$B_{7}^{\sigma }=-J_{11}^{\sigma }-J_{22}^{\sigma }-J_{33}^{\sigma }-J_{44}^{\sigma }$$

(A25) $$\begin{array}{ll} B_{6}^{\sigma } & =-J_{15}^{\sigma }-J_{12}^{\sigma }J_{21}^{\sigma }+J_{11}^{\sigma }J_{22}^{\sigma }-J_{26}^{\sigma }-J_{13}^{\sigma }J_{31}^{\sigma }-J_{23}^{\sigma }J_{32}^{\sigma }+J_{11}^{\sigma }J_{33}^{\sigma }+J_{22}^{\sigma }J_{33}^{\sigma }-J_{37}^{\sigma }-\\ & J_{14}^{\sigma }J_{41}^{\sigma }-J_{24}^{\sigma }J_{42}^{\sigma }-J_{34}^{\sigma }J_{43}^{\sigma }+J_{11}^{\sigma }J_{44}^{\sigma }+J_{22}^{\sigma }J_{44}^{\sigma }+J_{33}^{\sigma }J_{44}^{\sigma }-J_{48}^{\sigma } \end{array}$$

(A26) $$\begin{array}{ll} B_{5}^{\sigma } & =J_{15}^{\sigma }J_{22}^{\sigma }+J_{11}^{\sigma }J_{26}^{\sigma }+J_{13}^{\sigma }J_{22}^{\sigma }J_{31}^{\sigma }-J_{12}^{\sigma }J_{23}^{\sigma }J_{31}^{\sigma }-J_{13}^{\sigma }J_{21}^{\sigma }J_{32}^{\sigma }+J_{11}^{\sigma }J_{23}^{\sigma }J_{32}^{\sigma }+J_{15}^{\sigma }J_{33}^{\sigma }+J_{12}^{\sigma }J_{21}^{\sigma }J_{33}^{\sigma }-J_{11}^{\sigma }J_{22}^{\sigma }J_{33}^{\sigma }+\\ & J_{26}^{\sigma }J_{33}^{\sigma }+J_{11}^{\sigma }J_{37}^{\sigma }+J_{22}^{\sigma }J_{37}^{\sigma }+J_{14}^{\sigma }J_{22}^{\sigma }J_{41}^{\sigma }-J_{12}^{\sigma }J_{24}^{\sigma }J_{41}^{\sigma }+J_{14}^{\sigma }J_{33}^{\sigma }J_{41}^{\sigma }-J_{13}^{\sigma }J_{34}^{\sigma }J_{41}^{\sigma }-J_{14}^{\sigma }J_{21}^{\sigma }J_{42}^{\sigma }+J_{11}^{\sigma }J_{24}^{\sigma }J_{42}^{\sigma }+\\ & J_{24}^{\sigma }J_{33}^{\sigma }J_{42}^{\sigma }-J_{23}^{\sigma }J_{34}^{\sigma }J_{42}^{\sigma }-J_{14}^{\sigma }J_{31}^{\sigma }J_{43}^{\sigma }-J_{24}^{\sigma }J_{32}^{\sigma }J_{43}^{\sigma }+J_{11}^{\sigma }J_{34}^{\sigma }J_{43}^{\sigma }+J_{22}^{\sigma }J_{34}^{\sigma }J_{43}^{\sigma }+J_{15}^{\sigma }J_{44}^{\sigma }+J_{12}^{\sigma }J_{21}^{\sigma }J_{44}^{\sigma }-J_{11}^{\sigma }J_{22}^{\sigma }J_{44}^{\sigma }+\\ & J_{26}^{\sigma }J_{44}^{\sigma }+J_{13}^{\sigma }J_{31}^{\sigma }J_{44}^{\sigma }+J_{23}^{\sigma }J_{32}^{\sigma }J_{44}^{\sigma }-J_{11}^{\sigma }J_{33}^{\sigma }J_{44}^{\sigma }-J_{22}^{\sigma }J_{33}^{\sigma }J_{44}^{\sigma }+J_{37}^{\sigma }J_{44}^{\sigma }+J_{11}^{\sigma }J_{48}^{\sigma }+J_{22}^{\sigma }J_{48}^{\sigma }+J_{33}^{\sigma }J_{48}^{\sigma } \end{array}$$

(A27) $$\begin{array}{ll} B_{4}^{\sigma } & =J_{15}^{\sigma }J_{16}^{\sigma }+J_{13}^{\sigma }J_{26}^{\sigma }J_{31}^{\sigma }+J_{15}^{\sigma }J_{23}^{\sigma }J_{32}^{\sigma }-J_{15}^{\sigma }J_{22}^{\sigma }J_{33}^{\sigma }-J_{11}^{\sigma }J_{26}^{\sigma }J_{33}^{\sigma }+J_{15}^{\sigma }J_{37}^{\sigma }+J_{12}^{\sigma }J_{21}^{\sigma }J_{37}^{\sigma }-J_{11}^{\sigma }J_{22}^{\sigma }J_{37}^{\sigma }+\\ & J_{26}^{\sigma }J_{37}^{\sigma }+J_{14}^{\sigma }J_{26}^{\sigma }J_{41}^{\sigma }+J_{14}^{\sigma }J_{23}^{\sigma }J_{32}^{\sigma }J_{41}^{\sigma }-J_{13}^{\sigma }J_{24}^{\sigma }J_{32}^{\sigma }J_{41}^{\sigma }-J_{14}^{\sigma }J_{22}^{\sigma }J_{33}^{\sigma }J_{41}^{\sigma }+J_{12}^{\sigma }J_{24}^{\sigma }J_{33}^{\sigma }J_{41}^{\sigma }+J_{13}^{\sigma }J_{22}^{\sigma }J_{34}^{\sigma }J_{41}^{\sigma }-\\ & J_{12}^{\sigma }J_{23}^{\sigma }J_{34}^{\sigma }J_{41}^{\sigma }+J_{14}^{\sigma }J_{37}^{\sigma }J_{41}^{\sigma }+J_{15}^{\sigma }J_{24}^{\sigma }J_{42}^{\sigma }-J_{14}^{\sigma }J_{23}^{\sigma }J_{31}^{\sigma }J_{42}^{\sigma }+J_{13}^{\sigma }J_{24}^{\sigma }J_{31}^{\sigma }J_{42}^{\sigma }+J_{14}^{\sigma }J_{21}^{\sigma }J_{33}^{\sigma }J_{42}^{\sigma }-J_{11}^{\sigma }J_{24}^{\sigma }J_{33}^{\sigma }J_{42}^{\sigma }-\\ & J_{13}^{\sigma }J_{21}^{\sigma }J_{34}^{\sigma }J_{42}^{\sigma }+J_{11}^{\sigma }J_{23}^{\sigma }J_{34}^{\sigma }J_{42}^{\sigma }+J_{24}^{\sigma }J_{37}^{\sigma }J_{42}^{\sigma }+J_{14}^{\sigma }J_{22}^{\sigma }J_{31}^{\sigma }J_{43}^{\sigma }-J_{12}^{\sigma }J_{24}^{\sigma }J_{31}^{\sigma }J_{43}^{\sigma }-J_{14}^{\sigma }J_{21}^{\sigma }J_{32}^{\sigma }J_{43}^{\sigma }+J_{11}^{\sigma }J_{24}^{\sigma }J_{32}^{\sigma }J_{43}^{\sigma }+\\ & J_{15}^{\sigma }J_{34}^{\sigma }J_{43}^{\sigma }+J_{12}^{\sigma }J_{21}^{\sigma }J_{34}^{\sigma }J_{43}^{\sigma }-J_{11}^{\sigma }J_{22}^{\sigma }J_{34}^{\sigma }J_{43}^{\sigma }+J_{26}^{\sigma }J_{34}^{\sigma }J_{43}^{\sigma }-J_{15}^{\sigma }J_{22}^{\sigma }J_{44}^{\sigma }-J_{11}^{\sigma }J_{26}^{\sigma }J_{44}^{\sigma }-J_{13}^{\sigma }J_{22}^{\sigma }J_{31}^{\sigma }J_{44}^{\sigma }+\\ & J_{12}^{\sigma }J_{23}^{\sigma }J_{31}^{\sigma }J_{44}^{\sigma }+J_{13}^{\sigma }J_{21}^{\sigma }J_{32}^{\sigma }J_{44}^{\sigma }-J_{11}^{\sigma }J_{23}^{\sigma }J_{32}^{\sigma }J_{44}^{\sigma }-J_{15}^{\sigma }J_{33}^{\sigma }J_{44}^{\sigma }-J_{12}^{\sigma }J_{21}^{\sigma }J_{33}^{\sigma }J_{44}^{\sigma }+J_{11}^{\sigma }J_{22}^{\sigma }J_{33}^{\sigma }J_{44}^{\sigma }-J_{26}^{\sigma }J_{33}^{\sigma }J_{44}^{\sigma }-\\ & J_{11}^{\sigma }J_{37}^{\sigma }J_{44}^{\sigma }-J_{22}^{\sigma }J_{37}^{\sigma }J_{44}^{\sigma }+J_{15}^{\sigma }J_{48}^{\sigma }+J_{12}^{\sigma }J_{21}^{\sigma }J_{48}^{\sigma }-J_{11}^{\sigma }J_{22}^{\sigma }J_{48}^{\sigma }+J_{26}^{\sigma }J_{48}^{\sigma }+J_{13}^{\sigma }J_{31}^{\sigma }J_{48}^{\sigma }+J_{23}^{\sigma }J_{32}^{\sigma }J_{48}^{\sigma }-J_{11}^{\sigma }J_{33}^{\sigma }J_{48}^{\sigma }-\\ & J_{22}^{\sigma }J_{33}^{\sigma }J_{48}^{\sigma }+J_{37}^{\sigma }J_{48}^{\sigma } \end{array}$$

(A28) $$\begin{array}{ll} B_{3}^{\sigma } & =-J_{15}^{\sigma }J_{26}^{\sigma }J_{33}^{\sigma }-J_{15}^{\sigma }J_{22}^{\sigma }J_{37}^{\sigma }-J_{11}^{\sigma }J_{26}^{\sigma }J_{37}^{\sigma }-J_{14}^{\sigma }J_{26}^{\sigma }J_{33}^{\sigma }J_{41}^{\sigma }+J_{13}^{\sigma }J_{26}^{\sigma }J_{34}^{\sigma }J_{41}^{\sigma }-\\ & J_{14}^{\sigma }J_{22}^{\sigma }J_{37}^{\sigma }J_{41}^{\sigma }+J_{12}^{\sigma }J_{24}^{\sigma }J_{37}^{\sigma }J_{41}^{\sigma }-J_{15}^{\sigma }J_{24}^{\sigma }J_{33}^{\sigma }J_{42}^{\sigma }+J_{15}^{\sigma }J_{23}^{\sigma }J_{34}^{\sigma }J_{42}^{\sigma }+J_{14}^{\sigma }J_{21}^{\sigma }J_{37}^{\sigma }J_{42}^{\sigma }-\\ & J_{11}^{\sigma }J_{24}^{\sigma }J_{37}^{\sigma }J_{42}^{\sigma }+J_{14}^{\sigma }J_{26}^{\sigma }J_{31}^{\sigma }J_{43}^{\sigma }+J_{15}^{\sigma }J_{24}^{\sigma }J_{32}^{\sigma }J_{43}^{\sigma }-J_{15}^{\sigma }J_{22}^{\sigma }J_{34}^{\sigma }J_{43}^{\sigma }-J_{11}^{\sigma }J_{26}^{\sigma }J_{34}^{\sigma }J_{43}^{\sigma }-\\ & J_{15}^{\sigma }J_{26}^{\sigma }J_{44}^{\sigma }-J_{13}^{\sigma }J_{26}^{\sigma }J_{31}^{\sigma }J_{44}^{\sigma }-J_{15}^{\sigma }J_{23}^{\sigma }J_{32}^{\sigma }J_{44}^{\sigma }+J_{15}^{\sigma }J_{22}^{\sigma }J_{33}^{\sigma }J_{44}^{\sigma }+J_{11}^{\sigma }J_{26}^{\sigma }J_{33}^{\sigma }J_{44}^{\sigma }-\\ & J_{15}^{\sigma }J_{37}^{\sigma }J_{44}^{\sigma }-J_{12}^{\sigma }J_{21}^{\sigma }J_{37}^{\sigma }J_{44}^{\sigma }+J_{11}^{\sigma }J_{22}^{\sigma }J_{37}^{\sigma }J_{44}^{\sigma }-J_{26}^{\sigma }J_{37}^{\sigma }J_{44}^{\sigma }-J_{15}^{\sigma }J_{22}^{\sigma }J_{48}^{\sigma }-J_{11}^{\sigma }J_{26}^{\sigma }J_{48}^{\sigma }-\\ & J_{13}^{\sigma }J_{22}^{\sigma }J_{31}^{\sigma }J_{48}^{\sigma }+J_{12}^{\sigma }J_{23}^{\sigma }J_{31}^{\sigma }J_{48}^{\sigma }+J_{13}^{\sigma }J_{21}^{\sigma }J_{32}^{\sigma }J_{48}^{\sigma }-J_{11}^{\sigma }J_{23}^{\sigma }J_{32}^{\sigma }J_{48}^{\sigma }-J_{15}^{\sigma }J_{33}^{\sigma }J_{48}^{\sigma }-\\ & J_{12}^{\sigma }J_{21}^{\sigma }J_{33}^{\sigma }J_{48}^{\sigma }+J_{11}^{\sigma }J_{22}^{\sigma }J_{33}^{\sigma }J_{48}^{\sigma }-J_{26}^{\sigma }J_{33}^{\sigma }J_{48}^{\sigma }-J_{11}^{\sigma }J_{37}^{\sigma }J_{48}^{\sigma }-J_{22}^{\sigma }J_{37}^{\sigma }J_{48}^{\sigma } \end{array}$$

(A29) $$\begin{array}{ll} B_{2}^{\sigma } & =-J_{15}^{\sigma }J_{26}^{\sigma }J_{37}^{\sigma }-J_{14}^{\sigma }J_{26}^{\sigma }J_{37}^{\sigma }J_{41}^{\sigma }-J_{15}^{\sigma }J_{24}^{\sigma }J_{37}^{\sigma }J_{42}^{\sigma }-J_{15}^{\sigma }J_{26}^{\sigma }J_{34}^{\sigma }J_{43}^{\sigma }+J_{15}^{\sigma }J_{26}^{\sigma }J_{33}^{\sigma }J_{44}^{\sigma }+J_{15}^{\sigma }J_{22}^{\sigma }J_{37}^{\sigma }J_{44}^{\sigma }+J_{11}^{\sigma }J_{26}^{\sigma }J_{37}^{\sigma }J_{44}^{\sigma }-\\ & J_{15}^{\sigma }J_{26}^{\sigma }J_{48}^{\sigma }-J_{13}^{\sigma }J_{26}^{\sigma }J_{31}^{\sigma }J_{48}^{\sigma }-J_{15}^{\sigma }J_{23}^{\sigma }J_{32}^{\sigma }J_{48}^{\sigma }+J_{15}^{\sigma }J_{22}^{\sigma }J_{33}^{\sigma }J_{48}^{\sigma }+J_{11}^{\sigma }J_{26}^{\sigma }J_{33}^{\sigma }J_{48}^{\sigma }-J_{15}^{\sigma }J_{37}^{\sigma }J_{48}^{\sigma }-J_{12}^{\sigma }J_{21}^{\sigma }J_{37}^{\sigma }J_{48}^{\sigma }+\\ & J_{11}^{\sigma }J_{22}^{\sigma }J_{37}^{\sigma }J_{48}^{\sigma }-J_{26}^{\sigma }J_{37}^{\sigma }J_{48}^{\sigma } \end{array}$$

(A30) $$B_{1}^{\sigma }=J_{15}^{\sigma }J_{26}^{\sigma }J_{37}^{\sigma }J_{44}^{\sigma }+J_{15}^{\sigma }J_{26}^{\sigma }J_{33}^{\sigma }J_{48}^{\sigma }+J_{15}^{\sigma }J_{22}^{\sigma }J_{37}^{\sigma }J_{48}^{\sigma }+J_{11}^{\sigma }J_{26}^{\sigma }J_{37}^{\sigma }J_{48}^{\sigma }$$

(A31) $$B_{0}^{\sigma }=J_{15}^{\sigma }J_{26}^{\sigma }J_{37}^{\sigma }J_{48}^{\sigma }$$
